# ^1^H–^1^H Interatomic Distances in Paracetamol-Based Structures Unveiled by Double-Quantum NMR and DFT Calculations

**DOI:** 10.3390/molecules31101584

**Published:** 2026-05-09

**Authors:** Martins Balodis, Baltzar Stevensson, Debashis Majhi, Tra Mi Nguyen, Chaithanya Hareendran, Mattias Edén

**Affiliations:** Department of Chemistry, Stockholm University, SE-106 91 Stockholm, Sweden; martins.balodis@osi.lv (M.B.); baltzar.stevensson@su.se (B.S.); debashis@nitt.edu (D.M.); trantrami.nguyen@su.se (T.M.N.); chaithanya.hareendran@su.se (C.H.)

**Keywords:** acetaminophen, interatomic H–H distances, structure validation, double-quantum ^1^H–^1^H correlation NMR, hydrogen bonds

## Abstract

The crystal structures of monoclinic paracetamol, its cocrystal with oxalic acid (ParaOA), and its HCl monohydrate salt (ParaHCl) were refined by density functional theory (DFT) calculations and contrasted with the initial X-ray diffraction (XRD) structures. Two independent, but largely consistent, assessments were made: (*i*) comparisons between ^1^H and ^13^C chemical shifts obtained from magic-angle spinning (MAS) nuclear magnetic resonance (NMR) experiments and those predicted by plane-wave DFT calculations before and after geometry optimization; (*ii*) direct ^1^H–^1^H distance evaluations by a recently introduced NMR crystallography method that offers straightforward structure assessments due to interatomic-distance constraints from one double-quantum–single-quantum (2Q–1Q) ^1^H NMR correlation experiment. For both the ^1^H/^13^C chemical shift and ^1^H–^1^H distance assessments, the geometry-optimized ParaHCl structure offered a markedly better match than the initial XRD structure, while the XRD structure of paracetamol revealed excellent agreement with the NMR data, with only marginal improvements offered by the DFT optimization. The XRD-derived structure of ParaOA also agreed well with the NMR chemical shift/distance constraints: While the computed ^13^C chemical shifts showed better agreement with those from MAS NMR, slightly larger discrepancies were observed for the ^1^H chemical shifts and the ^1^H–^1^H distances. We also discuss the chemical shifts and present the first ^1^H and ^13^C MAS NMR-peak assignments for the ParaHCl and ParaOA structures.

## 1. Introduction

Access to reliable and efficient structure-determination tools is paramount for the development of new active pharmaceutical ingredients (APIs) in powdered forms. While X-ray diffraction (XRD) largely meets those requirements, it is well-known that it often provides unreliable proton positions even from single crystals, which is particularly problematic when studying weak noncovalent interactions, such as hydrogen [1,2,3,4,5,6,7] and tetrel [8,9] bonds in supramolecular aggregates, yet intermolecular interactions often govern the crystal structure, such as in salts and co-crystals [10,11] that are often employed for more efficient API administration. Although neutron diffraction offers accurate H positions, it is often not available for routine applications, whereas electron diffraction is prone to damage the structures of organic molecules. Moreover, diffraction-based techniques are normally of limited utility for probing disordered structures. Here, the utilization of ^1^H magic-angle-spinning (MAS) nuclear magnetic resonance (NMR) experimentation is an attractive alternative tool, largely thanks to its comparatively high NMR-signal sensitivity and applicability to well-ordered and amorphous materials [12,13,14,15,16]. Over the past two decades, solid-state ^1^H NMR utilization has increased significantly in pharmaceutical research due to the availability of steadily increasing MAS rates for suppressing the otherwise spectral-resolution-limiting effects of magnetic ^1^H–^1^H *dipolar* interactions, which are mediated directly through space and depend on the inverse cube of the interatomic ^1^H–^1^H distance [12,13,14,15].

Despite these advances, however, interferences among the vast number of mutually dipolar-coupled ^1^H spins in structures have severely compromised accurate de ${\mathrm{novo}}^{1}$H–^1^H distance determinations. Rather, the “NMR crystallography” branch has primarily relied on structure validations by contrasting experimental ^1^H, ^13^C, or ^15^N chemical shifts with those calculated from a crystal structure model refined by density functional theory (DFT) calculations [17,18,19,20,21,22,23,24]. While such procedures have indeed proven very powerful, they do not offer direct internuclear distance constraints. Several NMR crystallography studies utilizing advanced dipolar-based NMR techniques are nevertheless reported to date, most of which involve 2D NMR experimentation that recovers (“recouples”) dipolar interactions in pairs of nuclear spins that are otherwise largely or partially suppressed by MAS [12,13,14,15], where we henceforth focus only on homonuclear ^1^H–^1^H dipolar interactions/distances. Typically, these strategies rely on monitoring the 2D NMR-signal buildup of dipolar-driven ^1^H polarization transfers or double-quantum (2Q) coherences (2QC) [12,13,14,15,25,26,27,28,29,30,31,32,33] for progressively increasing dipolar recoupling periods to sample the spin dynamics, which in general demands acquiring several 2D NMR spectra and the subsequent integration of all relevant 2D NMR-peak intensities. Besides the experimental efforts, the extraction of precise ^1^H–^1^H internuclear distances is far from trivial for the strongly coupled multi-spin ^1^H systems of organic molecules. Current options rely either on time-consuming multi-spin spin-dynamics simulations that by necessity must be limited to analyzing only a few ^1^H sites (≲10) of the model structure to be validated [30,31,32] or by phenomenological approaches to approximate the spin dynamics [27,28,29]. At the price of limiting the information, however, selective dipolar recoupling of a specifically targeted spin pair may be employed [34,35,36].

Herein, we exploit direct ^1^H–^1^H distance constraints offered by a recently introduced NMR crystallography method that features some decisive advantages: It only involves analyses of one sole double-quantum–single-quantum (2Q–1Q) ^1^H correlation NMR spectrum [37,38], for which its integrated peak intensities may be used to readily validate any structure model obtained from experiments (e.g., XRD) or computational modeling. The method is general and has thus far been applied to probe ^1^H–^31^P and ^1^H–^1^H distances in the Ca phosphate mineral monetite [37], along with ^1^H–^31^P, ^1^H–^13^C and ^1^H–^1^H distances in phosphoserine and its Ca salt [38]. Despite its advantages, encompassing the alleviation of the “dipolar truncation” phenomenon that compromises determinations of long interatomic distances of two distant nuclear sites that are strongly dipolar-coupled to other neighbors [13,14,31], the NMR crystallography method has received little attention. To hopefully spread the utilization of this method, Section 2.5 outlines the procedure and discusses practical aspects specific for ^1^H–^1^H distance analyses, which partially complement and expand the information given in refs. [37,38].

**Figure 1:**
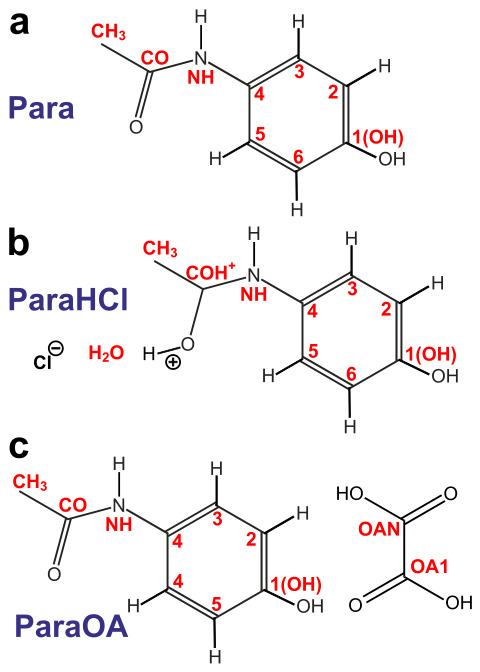
Molecular systems studied herein with their respective atom labeling of (**a**) paracetamol (“Para”), (**b**) paracetamol hydrochloride monohydrate salt (“ParaHCl”), and the (**c**) “ParaOA” cocrystal of paracetamol and oxalic acid (OA).

*N*-acetyl-*para*-aminophenol ($C_{8}H_{9}{\mathrm{NO}}_{2}$) is colloquially referred to as acetaminophen or paracetamol and constitutes one of the most commonly utilized APIs for reducing fever and pain. The mechanical properties of paracetamol powder make it difficult to compact into tablets, however, which has prompted the search for alternative formulations to enable efficient compaction, encompassing cocrystals [39] or salts [40]. Although salt formation is the most frequently employed route for API administration, it was not accomplished until recently for paracetamol [40]. Herein, we investigate the structures of monoclinic paracetamol, which is the most stable of the three polymorphs and is henceforth abbreviated as *Para* (Figure 1a), along with its hydrochloride monohydrate salt [40] (Figure 1b) and oxalic acid (OA) cocrystal [39] (Figure 1c), herein referred to as *ParaHCl* and *ParaOA*, respectively. Both facilitate tablet manufacture thanks to their higher plasticity [39,40]. We examine and refine three previously reported XRD-derived structures of Para [41], ParaHCl [40], and ParaOA [39]. They were refined by DFT calculations, whose improvements were assessed against the experimental 2Q–1Q ^1^H NMR constraints, as well as by the conventional approach of contrasting the NMR-derived ^1^H and ^13^C chemical shifts with those predicted by DFT calculations using the Gauge-Including Projector Augmented Wave (GIPAW) approach [17,18,19,20,21,22]. For the ParaHCl and ParaOA structures, the ^1^H/^13^C chemical shifts and their site assignments are, to the best of our knowledge, reported for the first time.

## 2. Results and Discussion

### 2.1. ^13^C and ^1^H NMR Spectra

**Figure 2:**
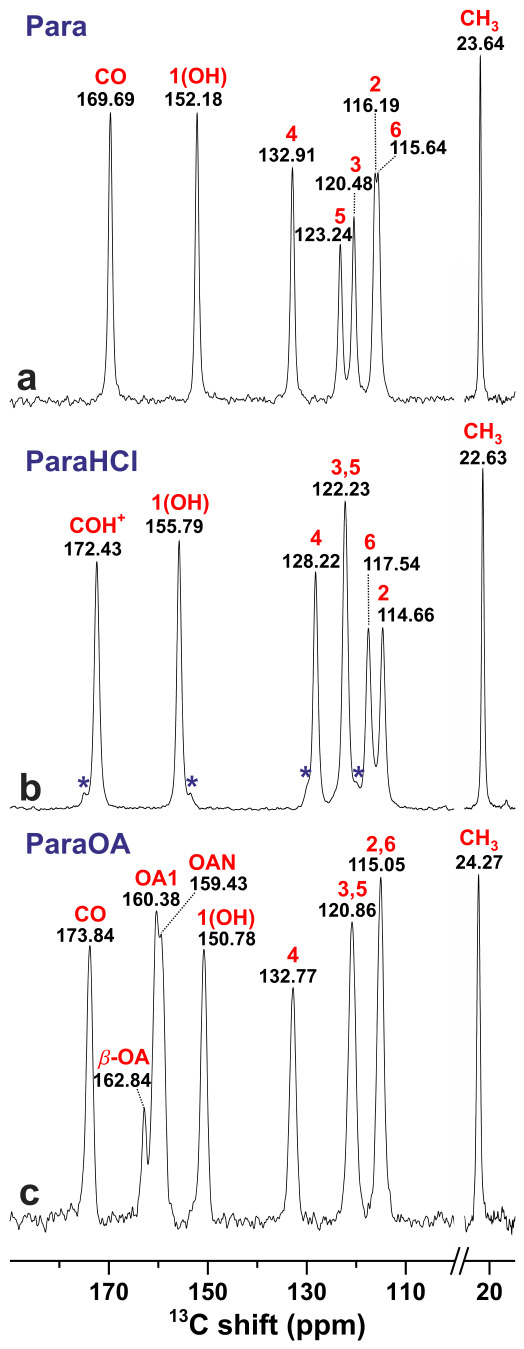
^13^C CPMAS NMR spectra acquired at 14.1 T and 12.00 kHz MAS from (**a**) Para, (**b**) ParaHCl, and the (**c**) ParaOA cocrystal. Each NMR peak was assigned to its respective ^13^C site identified in Figure 1 using DFT calculations. The chemical shift is specified by the number beneath (in ppm). The asterisks in (**b**) mark signals from a minor impurity attributed to bis(para)HCl; see Section 3.2. The resonance of a β-OA impurity is indicated in (**c**).

Figure 2 shows cross-polarization (CP) MAS (CPMAS) ^13^C NMR spectra recorded from the paracetamol-based samples, revealing overall well-resolved resonances across the entire spectral regions, encompassing the high-ppm ^13^**C**O range (>150 ppm) to the spectral region of aromatic ^13^C (100–135 ppm) and aliphatic ^13^**C**$H_{3}$ (30–35 ppm) sites. Not surprisingly, the very similar electronic environments of the aromatic ^13^C sites produce close ^13^C chemical shifts, where the degree of NMR signal overlap varies among the sites and specimens: While the two ^13^C3 and ^13^C5 resonances are well-resolved in the NMR spectrum from Para (Figure 2a), they overlap completely in the spectra recorded from the ParaHCl and ParaOA specimens (Figure 2b,c). Likewise, both ^13^C2/^13^C6 NMR peaks are superimposed in the NMR spectrum from ParaOA, whereas they are barely resolved in that from Para but well separated in the spectrum associated with the ParaHCl specimen.

Although Figure 2 supports the inference from powder XRD about nearly phase-pure specimens, minor impurities are evident from the ^13^C CPMAS NMR spectra of ParaHCl and ParaOA. Several small NMR peaks, such as those at 174.9 and 153.5 ppm, could be traced to the by powder XRD (PXRD) identified bis(Para)HCl phase; see Section 3.2. The ^13^C CPMAS NMR recorded from the ParaOA specimen reveals a minor signal at $\delta_{C}=162.9$ ppm (Figure 2c), and its origin is discussed below.

**Figure 3:**
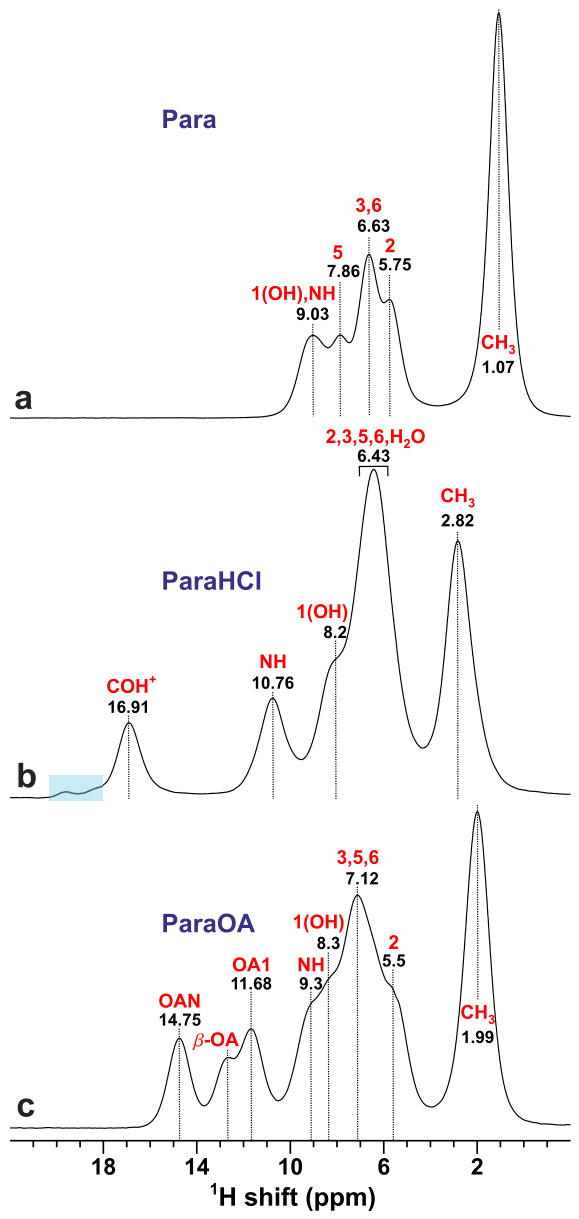
^1^H MAS NMR spectra recorded at 14.1 T and 60.0 kHz MAS from (**a**) Para, (**b**) ParaHCl, and the (**c**) ParaOA cocrystal. Each NMR peak is assigned to its respective ^1^H site shown in Figure 1 (Section 2.3), with the chemical shift at the peak maximum given by the number beneath (in ppm). The blue rectangle in (**b**) marks signals from a minor bis(para)HCl impurity. The resonance of a β-OA impurity is indicated in (**c**).

Figure 3 displays the ^1^H NMR spectra recorded at 14.1 T using a fast MAS rate of 60.0 kHz, which is sufficiently rapid for suppressing most of the otherwise substantial resonance broadening from homonuclear ^1^H–^1^H dipolar interactions [12,13,14,15]. All three spectra clearly resolve the $C^{1}H_{3}$ NMR signals found at the lowest chemical shifts (1–3 ppm), as well as all resonances from the OA-stemming ${\mathrm{COO}}^{1}$**H** sites and the acidic protons of ParaHCl appearing in the high-ppm range of >12 ppm. However, the aromatic protons resonating in the 5–9 ppm shift range are poorly resolved. The highest ^1^H chemical shifts ($\delta_{H}$) are observed for the 1($O^{1}$**H**) and $N^{1}$**H** sites ($\delta_{H}∼9$ ppm), which overlap significantly in the NMR spectra recorded from Para (Figure 3a) and ParaOA (Figure 3c) but are well-resolved in that from ParaHCl, which reveals a markedly higher $N^{1}$**H** shift at $\delta_{H}\approx 10.8$ ppm (Figure 3b). In contrast, the ^1^$H_{2}$O resonance of ParaHCl overlaps completely with those from the aromatic protons.

The presence of a minute impurity in the ParaHCl sample, as hinted from the ^13^C NMR spectrum (Figure 2b), is confirmed by the ^1^H MAS NMR counterpart of Figure 3b: Several small peaks (e.g., at 18.3 and 19.6 ppm) may be identified, which we attribute to MAS-induced ${\mathrm{ParaHCl\cdot H}}_{2}$O→bis(Para)HCl dehydration during prolonged sample spinning. Moreover, the ^1^H MAS NMR spectrum of ParaOA reveals well-resolved ${\mathrm{COO}}^{1}$**H** NMR peaks at 11.7 ppm (^1^**H**OA1) and 14.8 ppm (^1^**H**OAN) from the two distinct carboxy sites of the OA moiety, notwithstanding that both partially overlap with another NMR signal at $\delta_{H}\approx 12.7$ ppm (Figure 3c). The latter was unambiguously shown by 2Q–1Q ^1^H correlation NMR to stem from a separate phase (Section 2.2). It is attributed to the metastable β-OA polymorph [42], which apparently forms during fast MAS from the minor α-OA$\cdot 2H_{2}$O content of the ParaOA specimen identified by PXRD (Section 3.2). We are not aware of experimental ^13^C or ^1^H chemical shifts reported for the β-OA phase, but our DFT calculations predicted $\delta_{H}=13.7$ ppm and $\delta_{C}=163.2$ ppm. Incidentally, the latter shift is not only essentially identical to that of 162.8 ppm of Figure 3c but *also* to the shift of α-OA$\cdot 2H_{2}$O (163.1 ppm) [43], implying that the ^13^C NMR spectrum alone cannot discriminate between the β-OA and α-OA$\cdot 2H_{2}$O phases. Nonetheless, the ^1^H MAS and 2Q–1Q NMR spectra strongly suggest the presence of a minor β-OA component ($\delta_{H}=12.7$ ppm) but the absence of α-OA$\cdot 2H_{2}$O [44], which produces an NMR peak at a distinctly different shift of $\delta_{H}=16.6$ ppm [43].

### 2.2. 2Q–1Q ^1^H Correlation NMR Spectra

**Figure 4:**
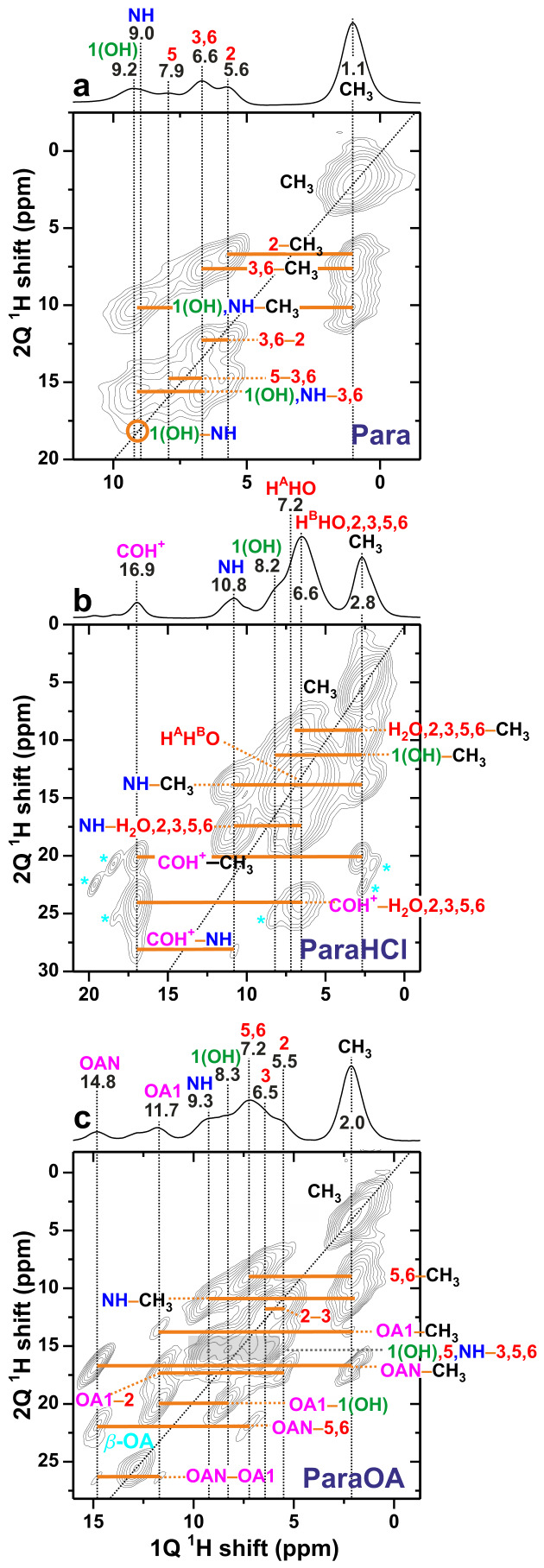
2Q–1Q ^1^H correlation NMR spectra recorded at 14.1 T and 60.00 kHz MAS from the (**a**) Para, (**b**) ParaHCl, and (**c**) ParaOA specimens by applying the BaBa recoupling sequence [45] for $\tau_{r}=16.67$ µs to excite/reconvert 2QC. Each 2Q–1Q ^1^H correlation peak is assigned to its pair of nearby ^1^H sites in each 2D NMR spectrum, for which projection along the horizontal 1Q dimension is shown at the top, with each NMR peak assigned and the number beneath specifying the chemical shift in ppm. The circle in (**a**) indicates the intermolecular correlation expected for N**H**⋯**1**(O**H**). The asterisks in (**b**) mark signals from a minor bis(para)HCl impurity, while the autocorrelation signal at $\delta_{2Q}=25.5$ ppm in (**c**) originates from a β-OA impurity. The grey rectangle in (**c**) marks the group of heavily overlapping 1(O**H**)/H5/N**H**⋯H3/H5/H6 correlations.

Figure 4 presents the 2Q–1Q ^1^H NMR spectra of the paracetamol-based specimens, recorded at fast MAS and a very short 2QC excitation interval of $\tau_{\mathrm{exc}}=16.67$ µs to enable a quantitative ^1^H–^1^H distance analysis [37,38] (Section 2.5). The spectra also offered valuable ^1^H NMR peak assignments, as discussed further in Section 2.3. A close proximity between two ^1^$H^{j}$ and ^1^$H^{k}$ sites that resonate at the corresponding chemical shifts $\delta_{H}^{j}$ and $\delta_{H}^{k}$ along the horizontal/direct “1Q dimension” ($\delta_{1Q}$) is evidenced by two 2D NMR peaks appearing at the spectral coordinates {$\delta_{2Q}^{jk}$, $\delta_{H}^{j}$} and {$\delta_{2Q}^{jk}$, $\delta_{H}^{k}$}, where the 2QC shift, $\delta_{2Q}^{jk}=\delta_{H}^{j}+\delta_{H}^{k}$, appears along the vertical/indirect “2Q dimension” ($\delta_{2Q}$) of the 2D NMR spectrum [12,13,14].

While spatial proximities between two crystallographically distinct proton sites with different chemical shifts generate two 2D NMR peaks for each 2QC, however, two nearby equivalent protons—such as the $C^{1}H_{3}$ protons of the paracetamol moiety—only produce one NMR peak at {$\delta_{2Q}$, $\delta_{1Q}$} = {2$\delta_{H}^{j}$, $\delta_{H}^{j}$} [12,13,14] (Figure 4). Notwithstanding the limited spectral resolution from the aromatic protons, correlations between the C$H_{3}$ sites with each of the N**H**, H2, H3, H5, and H6 protons are evident in the 2Q–1Q NMR spectrum from Para (Figure 4a). In the 2D NMR spectra obtained from the ParaHCl specimen shown in Figure 4b, the intense $C^{1}H_{3}$ autocorrelation signal partially overlaps with the 2Q correlation peak beneath that involves methyl and aromatic protons. Additional significant spectral overlaps also occur around the autocorrelation diagonal among signals from aromatic protons and the ^1^$H_{2}$O 2Q signal around {$\delta_{2Q}$, $\delta_{1Q}\}=\{$13.0, 6.5} ppm.

**Figure 5:**
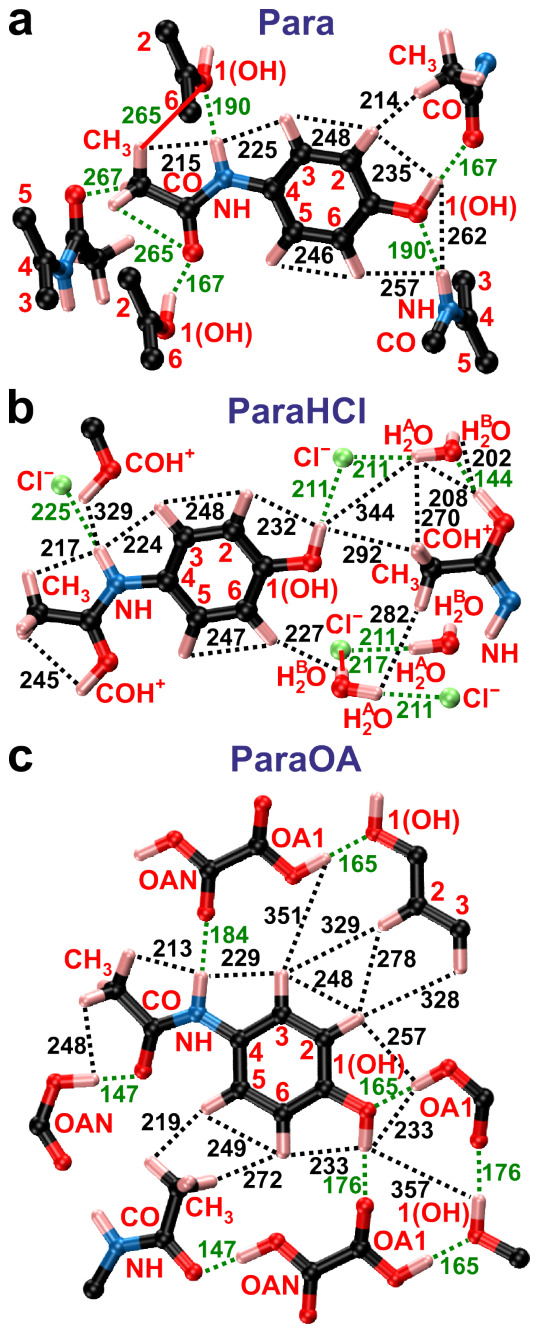
Fragments from the DFT-refined crystal structures of (**a**) Para, (**b**) ParaHCl, and (**c**) ParaOA. Each black dotted line marks the ^1^H–^1^H distance (in pm), while all green lines mark H-bond distances (pm) between the interconnected atom species.

Note that the 2Q–1Q ^1^H–^1^H signal associated with the *inter*molecular N**H**⋯1(O**H**) H bond indicated in Figure 5a is barely discernible in Figure 4a due to its expected low intensity and its overlap with the strong $N^{1}$**H**⋯H3/H6 correlation peak. The N**H**⋯1(O**H**) intermolecular contact of Para (Figure 5a) is superseded by one close *intra*molecular C$H_{3}\cdots$CO$H^{+}$ proximity and one longer intermolecular N**H**⋯CO$H^{+}$ contact in the ParaHCl structure (Figure 5b), as indeed evidenced by the respective 2QC correlations at $\delta_{2Q}=19.9$ ppm and $\delta_{2Q}=28.1$ ppm in Figure 4b. Likewise, the ParaOA structure involves two types of H bonds between the paracetamol and diacid moieties (Figure 5c): While that of N**H**⋯**H**OAN is comparatively long (386 pm) and is not detected in the 2Q–1Q NMR spectrum, the other involves the **H**OA1 and 1(O**H**) protons separated by 233 pm and manifested by the 2Q NMR peaks at $\delta_{2Q}=19.9$ ppm (Figure 4c). This ^1^H–^1^H correlation stems from two intermolecular H-bonds, 1**O**H⋯A1O**H** (165 pm) and 1O**H**⋯A1**O** (176 pm). The structural organization of the paracetamol and OA moieties also implies a comparatively short distance between the methyl protons and the **H**OAN molecular fragment (Figure 5c), as manifested by the two 2Q correlation signals at $\delta_{2Q}=16.8$ ppm. In contrast, the long C$H_{3}\cdots$**H**OA1 distance (346 pm) is revealed by the very weak 2Q correlation signals at $\delta_{2Q}=13.7$ ppm in Figure 4c.

The asterisks in Figure 4b mark the 2Q–1Q NMR peaks from the bis(para)HCl impurity (cf. Figure 3b). Notably, the absence of additional 2Q–1Q NMR correlations involving those protons and any ParaHCl-associated proton site unambiguously proves their presence in separate particles of the powder, as does the auto-correlation signal at $\delta_{2Q}=25.5$ ppm in Figure 4c, which stems from the β-OA impurity.

We conclude that the 2Q–1Q ^1^H NMR spectra agree qualitative well with the XRD-derived structures of refs. [39,40,41], whose DFT-refined counpterparts are depicted schematically in Figure 5. The quantitative aspects are discussed in Section 2.6.

### 2.3. NMR-Peak Assignments

**Figure 6:**
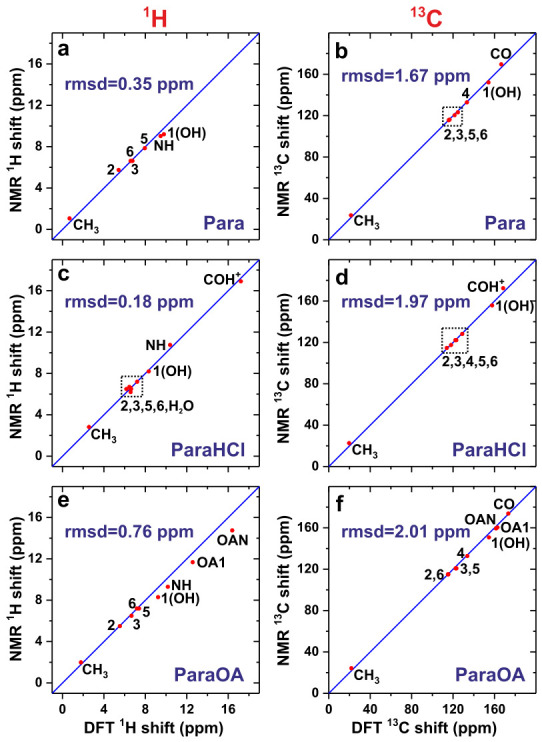
(**a**,**c**,**e**) ^1^H and (**b**,**d**,**f**) ^13^C chemical-shift correlations, where the results from NMR experiments (vertical axis) are plotted against those predicted by GIPAW/DFT (horizontal axis) for (**a**,**b**) Para, (**c**,**d**) ParaHCl, and (**e**,**f**) ParaOA. Each diagonal line represents the result of a perfect correlation, the deviation from which the as-indicated rmsd value was calculated.

Table 1 contrasts the ^1^H and ^13^C chemical shifts obtained from NMR with those calculated by the GIPAW/DFT of the XRD-derived crystal structure *before* (“XRD”) and *after* (“DFT”) geometry optimization by DFT. Figure 6 plots the experimental NMR-derived {$\delta_{C}$} and {$\delta_{H}$} data against their respective GIPAW/DFT predictions of the DFT-refined structures. Every ^13^C chemical shift from all three specimens was assigned to its respective C site by using the chemical shift/site mapping that minimized the difference between the experimental and DFT-derived chemical shifts, whereas NMR peak assignments to the ^1^H sites were assisted both by DFT calculations and the 2D NMR experiments, as illustrated below. To the best of our knowledge, the ^1^H/^13^C chemical shifts and their site assignments are reported for the first time for the ParaHCl and ParaOA structures.

The results for the monoclinic Para structure obtained from the DFT-based assignment of Table 1 corroborate the resonance assignments reported earlier by Zhou and Rienstra [46]. Both the {$\delta_{C}$} and {$\delta_{H}$} datasets herein and in ref. [46] are in excellent agreement, typically within 0.3 and 0.1 ppm for each ^13^C as well as ^1^H site, respectively, which are well within the experimental uncertainties anticipated from NMR work from two distinct laboratories. Notably, however, our DFT calculations also offer unambiguous discrimination *within* each pair of {2, 6} and {3, 5} sites for ^13^C and ^1^H, which could not be uniquely discriminated in previous HETCOR NMR studies [46,47,48]. Likewise, the DFT-assisted assignments of the chemical shifts with their C2/C6 and H2/H6 sites (Figure 5) resolve the 180° ambiguity of the benzene-ring orientation also for the ParaHCl and ParaOA structures.

As is evident from the ^1^H MAS NMR spectrum obtained from Para (Figure 3a), our assignments are only ambiguous for two pairs of protons, viz., H3/H6 and 1OH/NH. The latter two resonances were discriminated by the 2Q–1Q correlation NMR spectrum in Figure 4a, from which we deduced that the lower ^1^H chemical shift of $\delta_{H}=9.0$ ppm is associated with the amide proton, while that of $\delta_{H}=9.2$ ppm stems from the $O^{1}$**H** group, whose shift is slightly lower than $\delta_{H}=9.5$ ppm, as deduced in ref. [46] from ^1^${\mathrm{H\{}}^{13}$C} HETCOR experiments. *Only* the shifts of the H3/H6 pair could not be unambiguously assigned due to the too close values of the NMR shifts (Table 1), while the 2Q–1Q NMR spectrum also did not permit sufficient resolution for discriminating H3 from H6.

Most ^1^H chemical shifts extracted from the MAS NMR spectrum of ParaHCl were readily assigned by the DFT calculations, except for those of the aromatic H2–H6 sites and the two crystallographically distinct ^1^$H_{2}$O protons, all of which contribute to the essentially Gaussian NMR peak around 6.4 ppm (Figure 3b). The two distinct ^1^$H_{2}^{A}$O and ^1^$H_{2}^{B}$O resonances at 6.6 and 7.2 ppm (Table 1), respectively, were identified uniquely from the 2Q–1Q ^1^H NMR spectrum (Figure 4b), whereas ^13^${\mathrm{C\{}}^{1}$H} HETCOR NMR greatly assisted in the assignments of aromatic protons H2–H6. The H2 and H6 sites were identified from their respective chemical-shift correlations with their neighboring ^13^C sites, as deduced from the HETCOR 2D NMR spectrum (Figure S2). Unfortunately, the degenerate ^13^**C**3 and ^13^**C**5 chemical shifts did not admit unique assignments of the 2D HETCOR NMR peaks at $\delta_{H}=6.7$ ppm and $\delta_{H}=6.5$ ppm to the H3 and H5 sites. Each of those was attributed to H3 and H5 solely on the basis of their very close matches with the respective DFT-derived values of 6.62 and 6.46 ppm (Table 1).

For the ^1^H NMR-peak assignments of the ParaOA cocrystal (Figure 3c), all of the aromatic proton-stemming resonances and their assignments to sites H1–H6 were offered by the 2Q–1Q ^1^H NMR spectrum shown in Figure 4c. A close inspection of the ^1^H NMR peakshape in the 8–9 ppm range of Figure 3c confirms the presence of an NMR peak at ≈8.3 ppm, as also evidenced by the 2Q–1Q NMR spectrum of Figure 4c. The attribution of the NMR signal at $\delta_{H}=8.3$ ppm to $N^{1}$**H** was made from the DFT-derived $\delta_{H}$ value, noting that, although the DFT calculations overestimated 1($O^{1}$**H**) and $N^{1}$**H** chemical shifts by ≈0.9 ppm, they retain the order of $\delta_{H}($OH$)<\delta_{H}($NH) (Table 1).

### 2.4. DFT-Predicted Chemical Shifts Versus NMR

Table 1 also lists the *difference* between the calculated and experimental chemical shifts for each ^1^H or ^13^C site *j*:

(1a) $$\begin{array}{cc} \Delta_{H}^{j}\left[\alpha \right] & =\delta_{H}^{j}[\alpha ]-\delta_{H}^{j}[\mathrm{NMR}],\;\mathrm{with}\;\alpha =\left\{\mathrm{XRD},\;\mathrm{DFT}\right\}, \end{array}$$

(1b) $$\begin{array}{cc} \Delta_{C}^{j}\left[\alpha \right] & =\delta_{C}^{j}[\alpha ]-\delta_{C}^{j}[\mathrm{NMR}],\;\mathrm{with}\;\alpha =\left\{\mathrm{XRD},\;\mathrm{DFT}\right\}. \end{array}$$

As anticipated, initial DFT refinements of the H atom positions improve the subsequent GIPAW/DFT chemical-shift predictions, as reflected in overall lower root mean square deviations (rmsd) observed across each {$\Delta_{H}^{j}[$DFT]} and {$\Delta_{C}^{j}[$DFT]} set relative to their respective {$\Delta_{H}^{j}[$XRD]} and {$\Delta_{C}^{j}[$XRD]} counterparts (Table 1), except for the ^1^H shifts of ParaOA (*vide infra*). Figure 6 presents correlation plots of the experimental chemical shifts against the predictions from GIPAW/DFT.

Notwithstanding the well-known problems to obtain precise proton positions by XRD, they appear to be accurate in the XRD-derived structure of the monoclinic Para structure of ref. [41], as suggested by the minor improvements of the calculated ^13^C and ^1^H chemical shifts after DFT refinements of the H coordinates: The rmsd is reduced from 2.6 ppm (XRD) to 1.7 ppm (DFT) across the ensemble of ^13^C chemical shifts, whereas the rmsd for the predicted ^1^H chemical shifts of Para revealed only a marginal reduction from 0.38 ppm (XRD) to 0.35 ppm (DFT). We also performed DFT optimizations and GIPAW/DFT calculations of a previously reported structure deduced from neutron diffraction [49]. However, both the ^13^C and ^1^H chemical shifts of the methyl group deviated significantly more to the NMR shifts than those of the XRD structure of ref. [41].

Significant improvements in the $\delta_{H}$ and $\delta_{C}$ predictions resulted upon the DFT optimization of the XRD-derived ParaHCl structure [40]. It appears to have several questionable H-atom coordinates, as mirrored in the significant reduction in the rmsd with respect to the experimental $\delta_{H}$ data from 6.0 ppm to only 0.18 ppm after DFT optimization, which is also markedly lower than any of the DFT-refined Para and ParaOA structures (Figure 6). The main improvements are observed for the aromatic ^1^**H**5 chemical shift along with those of the $H_{2}$O molecule. Drastic improvements also resulted for the $\delta_{C}$ predictions of ParaHCl: A substantial rmsd value of 9.7 ppm of the XRD structure was reduced to 2.0 ppm after DFT optimization. However, the unusually large rmsd for the ^13^C chemical shifts originates primarily from the staggering error of $\Delta_{C}[$XRD]=−25 ppm observed for the ^13^**C**$H_{3}$ group (Table 1). Nonetheless, even when omitting that result from the rmsd evaluation, a sizable rmsd of 4.5 ppm remains for the {$\Delta_{C}^{j}[$DFT]} set.

The DFT refinements of the ParaOA structure only improved the agreement between the calculated and experimental ^13^C shifts slightly, while the ^1^H counterparts revealed an *even larger* discrepancy of rmsd = 0.76 ppm compared to the shifts predicted from the initial XRD structure of rmsd = 0.58 ppm (Table 1). These apparent discrepancies are resolved by the ^1^H–^1^H distance analysis that confirmed a slightly worse agreement of the DFT structure relative to the NMR experiments (Section 2.6.4). Besides potential bearings from inaccuracies in the reported unit-cell parameters (see Section 3.5), we have no obvious explanation for the larger deviations observed after DFT refinement, which concern primarily the H sites involved in H bonding, i.e., the 1O**H**, N**H** and the two COO**H** sites of OA. Yet, these discrepancies remained significant and of comparable magnitude among several DFT optimizations with different functionals and input parameters.

However, the unexpected finding for ParaOA has an important message, namely the utility of contrasting chemical-shift predictions before and after geometry optimization, which (surprisingly) is normally not evaluated in published studies. Such evaluations offer a diagnostic tool by verifying the (degree of) improvements following the geometry optimization by DFT, where only minor improvements in accordance with the experimental chemical shifts confirm the good quality even of the input structure (as for Para herein), while an unexpected increased discrepancy might suggest problems related to the DFT calculations.

### 2.5. ^1^H–^1^H Distance Analysis Procedure

This section recapitulates the interatomic-distance-based NMR crystallography method of refs. [37,38], which is utilized and commented further herein. The dipolar coupling constant of two given structural sites *m* and *n* of one ^1^H^*j*^–^1^H^*k*^ pair with an internuclear distance $r_{mn}^{jk}$ is given by [12,13,14]

(2) $$b\left(H_{m}^{j}{\text{–}H}_{n}^{k}\right)=K_{\mathrm{HH}}{\left(r_{mn}^{jk}\right)}^{-3},\;\;\mathrm{with}\;K_{\mathrm{HH}}=-\mu_{0}\hbar \gamma_{H}^{2}/8\pi^{2}\;\;[\mathrm{units}\;\mathrm{of}\;\mathrm{Hz}],$$

where *j* and *k* refer to protons selected from two formally equal or distinct functional groups. Here, $\mu_{0}$ is the permeability of the vacuum, and $\gamma_{H}$ is the magnetogyric ratio of ^1^H [12,13,14]. The expression for the squared *effective dipolar coupling constant*, $b_{\mathrm{eff}}^{2}$($H^{j}$–$H^{k}$), depends on whether the two proton sites are chemically *equivalent* (which may stem from a crystallographic equivalence or rapid molecular dynamics) or *non*equivalent (j≠k):

(3) $$b_{\mathrm{eff}}^{2}\left(H^{j}{\text{–}H}^{k}\right)=\left\{\begin{array}{c} \sum_{m=1}^{M_{j}}\sum_{n=1}^{M_{k}}b^{2}\left(H_{m}^{j}{\text{–}H}_{n}^{k}\right),\;\mathrm{for}\;j\neq k\\ \sum_{m<n}^{M_{j}}b^{2}\left(H_{m}^{j}{\text{–}H}_{n}^{j}\right),\;\mathrm{for}\;j=k. \end{array}\right..$$

Owing to its ($r_{mn}^{jk}{)}^{-6}$ dependence, the $b_{\mathrm{eff}}^{2}(H^{j}$–$H^{k})$ value is predominantly dictated by the shortest $H_{m}^{j}$–$H_{n}^{k}$ distances. The choices of $M_{j}$ and $M_{k}$ are somewhat arbitrary, where our evaluations employed all distances up to 1.0 nm to reach well-converged $b_{\mathrm{eff}}^{2}(H^{j}$–$H^{k})$ values.

Note that the 2Q–1Q NMR experiments involve active dipolar recoupling/restoration by an rf-pulse sequence that generates an effective 2Q Hamiltonian with a scaled dipolar coupling constant [12,13,14,45,50], $\kappa b(H_{m}^{j}$–$H_{n}^{k})$, for which its precise value $\left|\kappa \right|<1$ is equal for all dipolar interactions but this becomes immaterial because the scaling factor is canceled in the structural analysis (e.g., see Equations (4) and (6) below). Provided that the 2Q–1Q correlation ^1^H NMR spectrum is acquired with a sufficiently *short* 2QC excitation period, then $b_{\mathrm{eff}}^{2}(H^{j}$–$H^{k})$ is directly proportional to the integrated 2D NMR spectral intensity generated by all $H_{m}^{j}$–$H_{n}^{k}$ pairs in the structure. “Sufficiently short” means, in practice, that every integrated 2D NMR spectral intensity from a $H_{m}^{j}$–$H_{n}^{k}$ pair should remain ≲1/3 of the maximum intensity observed from a 2D spectrum recorded with a longer $\tau_{\mathrm{exc}}$ value. We define the *fractional intensity*, $f_{\mathrm{NMR}}(H^{j}$–$H^{k}$), as the ratio between the integrated 2D NMR-peak intensity, $I(H^{j}$–$H^{k})$, and the total integrated 2D NMR spectral intensity ($I_{\mathrm{tot}}$) [37,38]:

(4) $$f_{\mathrm{NMR}}{(H}^{j}{\text{–}H}^{k})=I\left(H^{j}{\text{–}H}^{k}\right)/I_{\mathrm{tot}},$$

The {$f_{\mathrm{NMR}}$($H^{j}$–$H^{k}$)} set is normalized to unity:

(5) $$\sum_{j,k}f_{\mathrm{NMR}}{(H}^{j}{\text{–}H}^{k})=1.$$

Each $I(H^{j}$–$H^{k})$ value was determined from the average 2D NMR peak volume across several independent integrations, each obtained over a rectangular frequency domain.

The value of $f_{\mathrm{NMR}}$($H^{j}$–$H^{k})$ extracted from the 2Q–1Q ^1^H NMR experiment may be contrasted with that calculated for any structural model derived by experiments or modeling [37,38]:

(6) $$f_{\alpha }\left(H^{j}{\text{–}H}^{k}\right)=b_{\mathrm{eff}}^{2}\left(H^{j}{\text{–}H}^{k}\right)/b_{\mathrm{eff}}^{2}\left(\mathrm{tot}\right),\;\mathrm{with}\;\alpha =\{\mathrm{DFT},\mathrm{XRD}\},$$

where $b_{\mathrm{eff}}^{2}($tot) is the squared total dipolar interaction between *all* ^1^H–^1^H pair interactions in the structure:

(7) $$b_{\mathrm{eff}}^{2}\left(\mathrm{tot}\right)=\sum_{j,k}b_{\mathrm{eff}}^{2}\left(H^{j}{\text{–}H}^{k}\right).$$

Herein, we consider both the XRD-derived crystal structures for the paracetamol-based structures and their DFT-optimized counterparts, where a comparison of the results from Equations (4) and (6) offers a direct quantitative assessment of the validity of the evaluated structure model (Section 2.5.2).

#### 2.5.1. Effective ^1^H–^1^H Distances

An “effective” interatomic distance, $r_{\mathrm{eff}}^{\alpha }(H^{j}$–$H^{k}$), may be calculated for each $H^{j}$–$H^{k}$ pair in a structure according to [37,38]:

(8) $$r_{\mathrm{eff}}^{\alpha }\left(H^{j}{\text{–}H}^{k}\right)={\left(\frac{M_{jk}K_{\mathrm{HH}}^{2}}{b_{\mathrm{eff}}^{2}\left(H^{j}{\text{–}H}^{k}\right)}\right)}^{1/6},\;\mathrm{with}\;\alpha =\{\mathrm{XRD},\mathrm{DFT}\},$$

where $K_{\mathrm{HH}}$ is given by Equation (2), and $M_{jk}$ is the multiplicity of the set of largest dipolar coupling constants $b^{2}(H_{m}^{j}$–$H_{n}^{k})$ out of all terms in the sum of Equation (3) [37,38]. For instance, $M_{jk}=3$ for the methyl group of paracetamol. Each value of $r_{\mathrm{eff}}^{\mathrm{XRD}}(H^{j}$–$H^{k})$ or $r_{\mathrm{eff}}^{\mathrm{DFT}}(H^{j}$–$H^{k})$ extracted via Equation (8) is typically very similar to the shortest interatomic distance within the ${\mathrm{\{H}}_{m}^{j}$–$H_{n}^{k}$} set encountered in the respective XRD- and DFT-derived structure. The exact $r_{\mathrm{eff}}(H^{j}$–$H^{k})$ value may be slightly longer or shorter [37,38], however, because it depends on the precise number of proton pairs included in the sum of Equation (3) to reach convergence.

Likewise, an effective $H^{j}$–$H^{k}$ distance may be obtained from the {$f_{\mathrm{NMR}}$($H^{j}$–$H^{k})$} data extracted from the 2Q–1Q ^1^H NMR experiment provided that it is evaluated against a given “reference” structure, for which its $b_{\mathrm{eff}}^{2}($tot) result is effectively equated with $I_{\mathrm{tot}}$ of Equation (4) such that each fractional intensity may be converted into a $b_{\mathrm{eff}}^{2}(H^{j}$–$H^{k})$ value according to [37,38]

(9) $$b_{\mathrm{eff}}^{2}\left(H^{j}{\text{–}H}^{k}\right)=f_{\mathrm{NMR}}{(H}^{j}{\text{–}H}^{k})b_{\mathrm{eff}}^{2}\left(\mathrm{tot}\right).$$

Inserting the NMR-derived $b_{\mathrm{eff}}^{2}(H^{j}$–$H^{k})$ value into Equation (8) then yields the associated effective distance $r_{\mathrm{eff}}^{\mathrm{NMR}}(H^{j}$–$H^{k}),$ which we stress should not be overinterpreted because the precise value is biased to the particular reference structure (herein, XRD or DFT) from which $b_{\mathrm{eff}}^{2}($tot) was calculated and used in Equation (9).

#### 2.5.2. Structure Validation

For a straightforward validation and assessment of the quality of the XRD- and DFT-derived structures, Table 2 lists the respective correlation coefficient ($R^{2})$ of each set of fractional intensities {$f_{\mathrm{XRD}}(H^{j}$–$H^{k})$} and $f_{\mathrm{DFT}}(H^{j}$–$H^{k})$ calculated from Equation (6) with respect to the set of experimental fractional NMR intensities obtained from Equation (4):

(10) $$R^{2}\left(\alpha \right)=1-N_{jk}^{-1}\sum_{j,k}{\left[f_{\alpha }{(H}^{j}{\text{–}H}^{k})-f_{\mathrm{NMR}}{(H}^{j}{\text{–}H}^{k})\right]}^{2},\;\mathrm{with}\;\alpha =\{\mathrm{XRD},\mathrm{DFT}\},$$

Here, $N_{jk}=N_{jk}$Var$\{f_{\mathrm{NMR}}$($H^{j}$–$H^{k})\}$, where $N_{jk}$ is the number of distinct *j*–*k* pairs entering the sum for the given Para, ParaHCl, or ParaOA structure, and Var$\{f_{\mathrm{NMR}}$($H^{j}$–$H^{k})\}$ is the variance of the $\{f_{\mathrm{NMR}}$($H^{j}$–$H^{k})\}$ set. Whenever the 2D NMR spectral resolution did not admit sufficient signal-separation among distinct $H^{j}$–$H^{k}$ pairs, they were grouped together (Table 2), which was also accounted for in the accompanying XRD and DFT analyses via Equations (6) and (7). Note that despite limiting the statistics by offering fewer experimental distance constraints, validation of the model structure against the experiment is nevertheless perfectly feasible. Indeed, provided that a decent number of distance constraints (5–10) is available, the accuracy of {$f_{\mathrm{NMR}}$($H^{j}$–$H^{k})$} data from larger groups of H sites is normally higher than expanding the distance set using additional but more uncertain integrated intensities from strongly overlapping NMR signals.

**Figure 7:**
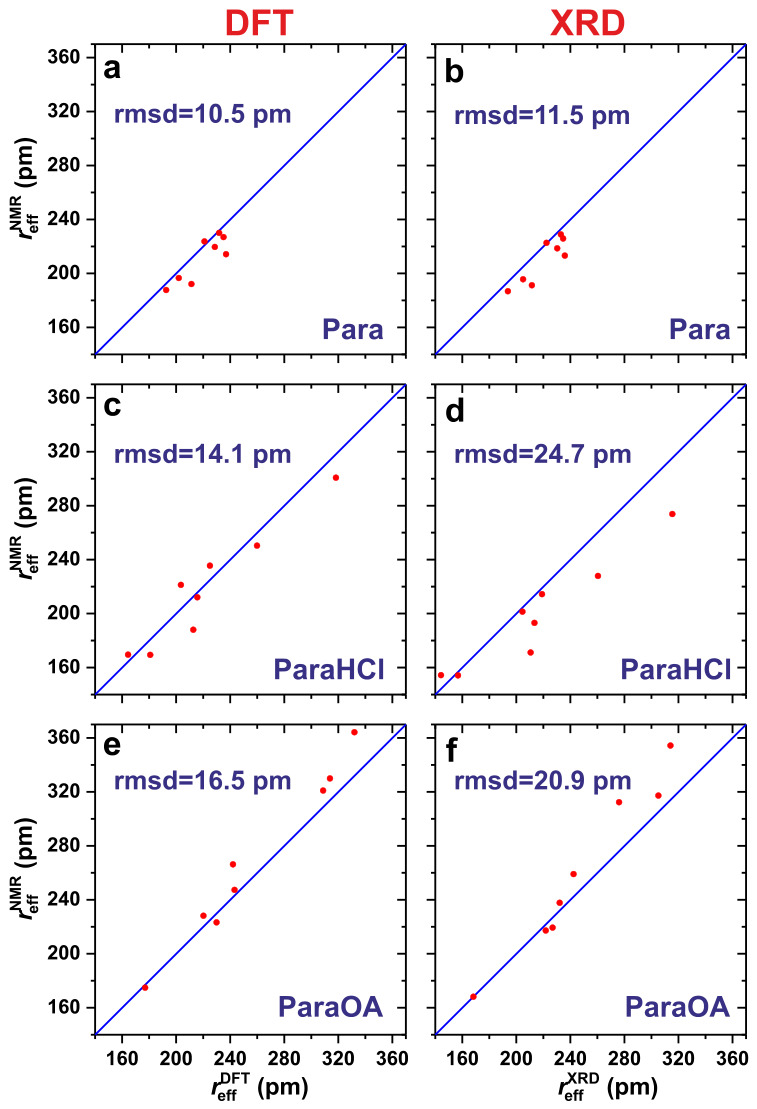
Correlation of the NMR-derived effective ^1^$H^{j}$–^1^H^*k*^ distance [$r_{\mathrm{eff}}^{\mathrm{NMR}}(H^{j}$–$H^{k})$] plotted against those calculated from the crystal structures (**a**,**c**,**e**) after DFT optimization [$r_{\mathrm{eff}}^{\mathrm{DFT}}(H^{j}$–$H^{k})$] of the initial (**b**,**d**,**f**) XRD structures [$r_{\mathrm{eff}}^{\mathrm{XRD}}(H^{j}$–$H^{k})$] of (**a**,**b**) Para [41], (**c**,**d**) ParaHCl [40], and (**e**,**f**) ParaOA [39]. The rmsd deviation is provided in each plot.

The effective-distance sets {$r_{\mathrm{eff}}^{\mathrm{XRD}}$($H^{j}$–$H^{k}$)} and {$r_{\mathrm{eff}}^{\mathrm{DFT}}$($H^{j}$–$H^{k}$)} generated for each XRD-/DFT-derived structure of Para, ParaHCl, and ParaOA are listed in Table 2, together with the NMR-derived counterpart, {$r_{\mathrm{eff}}^{\mathrm{NMR}}$($H^{j}$–$H^{k}$)}. The associated correlation plots are shown in Figure 7. For every $H^{j}$–$H^{k}$ pair in each structure, Table 2 also presents the deviation between the effective distance of each XRD and DFT structure, $r_{\mathrm{eff}}^{\mathrm{XRD}}$ and $r_{\mathrm{eff}}^{\mathrm{DFT}}$, relative to the value of $r_{\mathrm{eff}}^{\mathrm{NMR}}$,

(11) $$\Delta r_{\mathrm{eff}}^{\alpha }\left(H^{j}{\text{–}H}^{k}\right)=r_{\mathrm{eff}}^{\alpha }\left(H^{j}{\text{–}H}^{k}\right)-r_{\mathrm{eff}}^{\mathrm{NMR}}\left(H^{j}{\text{–}H}^{k}\right),\;\mathrm{with}\;\alpha =\{\mathrm{XRD},\mathrm{DFT}\},$$

as well as the rmsd over the entire set {$\Delta r_{\mathrm{eff}}^{\alpha }(H^{j}$–$H^{k})$} for each XRD/DFT method and Para, ParaHCl, or ParaOA structure. Note that although the $R^{2}\left(\alpha \right)$ and rmsd{$\Delta r_{\mathrm{eff}}^{\alpha }$} results normally provide identical conclusions, in the rare scenarios of discrepancies (Section 2.6.4), we recommend using the more accurate $R^{2}\left(\alpha \right)$ value calculated from Equation (10).

Notably, the distance analysis may produce significant discrepancies between the two $r_{\mathrm{eff}}^{\mathrm{NMR}}$($H^{j}$–$H^{k})$ values extracted by reference to each XRD or DFT structure. This occurs when there is a large deviation between the $f_{\mathrm{NMR}}$($H^{j}$–$H^{k})$ value and that from either XRD [$f_{\mathrm{XRD}}$($H^{j}$–$H^{k})$] or DFT [$f_{\mathrm{DFT}}$($H^{j}$–$H^{k})]$ (or both), which is typically reflected in comparatively low $R^{2}($XRD) and/or $R^{2}($DFT) values of ≲0.90. If $f_{\mathrm{NMR}}$($H^{j}$–$H^{k})$ is *larger* (*smaller*) than that of $f_{\alpha }(H^{j}$–$H^{k})$ calculated from the XRD/DFT structure, then the $r_{\mathrm{eff}}^{\mathrm{NMR}}(H^{j}$–$H^{k})$ value will be *shorter* (*longer*) than the effective distance of the model/reference structure, signifying that the latter distance is *over*estimated (*under*estimated). Significant discrepancies were only observed for the ParaHCl structure model. Below, we only discuss the precise effective-distances values of the XRD and DFT structures, while the $r_{\mathrm{eff}}^{\mathrm{NMR}}$ data listed in Table 2 only serves the purpose of giving $\Delta r_{\mathrm{eff}}^{\alpha }(H^{j}$–$H^{k})$ assessments via Equation (11), while indicating if the ^1^H–^1^H distance consistent with the NMR experiment is longer or shorter than that of the XRD/DFT structure.

### 2.6. ^1^H–^1^H Distance Results

#### 2.6.1. Results on Para

While a close inspection of the 2Q–1Q ^1^H NMR spectrum of Para (Figure 4a) enabled discrimination of the close chemical shifts of 9.0 ppm and 9.2 ppm of the N**H** and **1**(O**H**) sites, respectively, the significant overlap of all correlation signals from those sites prevented the ^1^H–^1^H distance analysis of each individual site. Hence, those two proton sites were grouped together in Table 2, as were the H3 and H6 protons with equal chemical shifts at $\delta_{H}=6.6$ ppm (Table 1).

Along the findings of marginally improved GIPAW/DFT chemical-shift predictions upon structure refinement by DFT, essentially equal discrepancies are observed for each {$r_{\mathrm{eff}}^{\mathrm{XRD}}$} and {$r_{\mathrm{eff}}^{\mathrm{DFT}}$} dataset relative to the NMR counterpart, both yielding rmsd values of ≈11 pm [Equation (11)] and very high and equal correlation coefficients of $R^{2}=0.98$ from Equation (10); see Table 2 and Figure 7. Altogether, this confirms the unusually high accuracy of the H positions of the XRD structure of ref. [41]. The two largest discrepancies in any individual effective distance are observed for the C$H_{3}\cdots$**H**3/**H**6 pair with $\Delta r_{\mathrm{eff}}^{\mathrm{XRD}}=20$ pm, along with the H2⋯H3/H6 pair with $\Delta r_{\mathrm{eff}}^{\mathrm{XRD}}=23$ pm, none of which was reduced by the DFT optimization (Table 2).

We now focus on the effective distances of various proton pairs, $r_{\mathrm{eff}}(H^{j}$–$H^{k})$. The shortest interatomic distances encountered in each DFT-refined structure of Para, ParaHCl, or ParaOA are given in Figure 5. Carefully distinguish the individual interatomic distance of a selected proton pair, $r(H_{m}^{j}$–$H_{n}^{k}$), from its “effective” counterpart, $r_{\mathrm{eff}}(H^{j}$–$H^{k}$) [Equation (8)], which is the *aggregate* value of the entire {$r(H_{m}^{j}$–$H_{n}^{k}$)} distance set encountered in the underlying structure model. As expected, the methyl protons of Para manifest the shortest effective distance of all pairs (≈177 pm), which is derived from the three {176, 178, 179} pm distances of the DFT-refined structure. The second shortest effective distance occurs for the group of C$H_{3}\cdots$N**H/**1O**H** pairs with $r_{\mathrm{eff}}^{\mathrm{DFT}}$ = 193 pm. By inspecting the DFT structure of Para, one may conclude that most 2Q NMR peak intensities at $\delta_{2Q}=10$ ppm (Figure 4a) stem from the intramolecular C$H_{3}\cdots$N**H** contact of 215 pm (Figure 5a), while the intermolecular C$H_{3}\cdots$1O**H** distance is much longer (266 pm) and thereby contributing markedly less to the 2D NMR spectral intensity. We note that the 2D NMR peak intensities scale as ($r_{mn}^{jk}{)}^{-6}$; see Section 2.5. All remaining proton-pair effective distances listed in Table 2 are longer than 200 pm, most of which involve either the methyl protons or the three-bond contacts of the aromatic protons, such as H2⋯H3 and H5⋯H6. What is notable is also the unexpectedly short intermolecular H5⋯H5 effective distance of ≈221 pm, which stems from the preferred relative orientations of two neighboring paracetamol molecules in the crystal structure.

#### 2.6.2. Bearings from Methyl-Group Dynamics

The dipolar interaction is sensitive to molecular dynamics: It averages to zero for (infinitely) fast MAS or rapid unrestricted molecular dynamics, while anisotropic motions reduce the (effective) dipolar coupling constant to a finite value [12,13,14,31]. It is well-known that the rapid rotational motion of *X*–${\mathrm{CH}}_{3}$ moieties around the *X*–C axis—or three-site jumps between the protons—scales each ^1^H–^1^H interaction to an effective value of −1/2 relative to that of the dipolar coupling constant for an immobile ${\mathrm{CH}}_{3}$ group [12,31]. Hence, accurate interatomic-distance analyses of the integrated 2Q–1Q NMR intensities must account for molecular motion. Unfortunately, that is only straightforward in the two limiting cases when the dynamics is either rapid or absent altogether. The crystal structure governs the presence/absence of rotation of the ${\mathrm{CH}}_{3}$ group, where, for instance, Zhou and Rienstra [46] observed variable degrees of motional averaging among the methyl moieties of ibuprofen.

The methyl-group rotation (or three-site jump) in the Para structure appears to be *neither* absent nor free but is presumably strongly restricted, as suggested by the slightly lower fractional intensity obtained from NMR ($f_{\mathrm{NMR}}$) of the ^1^H–^1^H interactions relative to the $f_{\mathrm{XRD}}$ and $f_{\mathrm{DFT}}$ counterparts calculated from the atomic coordinates in the absence of any dynamics (Table 2). The discrepancy is nonetheless much lower than that for a rapid free rotation or three-site jump, which would scale the static $b_{\mathrm{eff}}^{2}(H^{j}$–$H^{k})$ value by the factor (1/2)^2^ = 1/4 As depicted in Figure 5a, all three methyl protons in Para are H-bonded to nearby O atoms, which likely hinders their mobility. Notably, a significant ${\mathrm{CH}}_{3}$ mobility may be ruled out from the markedly reduced $R^{2}($DFT)=0.74 value resulting if instead assuming a fast three-site jump of the H sites when evaluating Equations (6) and (10).

In contrast, there are no H bonds involving ${\mathrm{CH}}_{3}$ in the ParaHCl structure (Figure 5b), while only two of the ${\mathrm{CH}}_{3}$ protons of ParaOA are involved in H bonding (Figure 5c). Indeed, the markedly lower $f_{\mathrm{NMR},}$ $f_{\mathrm{XRD}},$ and $f_{\mathrm{DFT}}$ values relative to those for Para (Table 2) suggest highly mobile methyl protons in both ParaHCl and ParaOA structures, for which our distance analyses assumed a rapid three-site jump model of the methyl group. Note that the effective dipolar interaction between the three C$H_{3}$ sites and an *external* H site is equal in the two scenarios of three static methyl protons and a rapid three-site jump model.

#### 2.6.3. Results of ParaHCl

The overall stronger NMR-signal overlap of the 2Q–1Q NMR signals from the aromatic protons of both ParaHCl and ParaOA structures required more extensive groupings of the integrated signal intensities shown in Figure 4b,c and the distance sets included in the sum of Equation (6). As expected from the significant improvements of the $\delta_{H}$ data from GIPAW/DFT calculations upon DFT optimization of the ParaHCl structure, the latter model agrees better with the ^1^H–^1^H distance constraints from NMR, with the correlation coefficient increasing from $R^{2}$(XRD)=0.82 to $R^{2}$(DFT)=0.90, while the rmsd across the distance sets reduced from 25 pm to 14 pm (Table 2 and Figure 7).

Notwithstanding a clear improvement upon DFT optimization, however, the structure still deviates markedly to the NMR experiment. Owing to the insufficient NMR-signal discrimination of the central region of the 2Q–1Q ^1^H NMR spectrum recorded from ParaHCl (Figure 4b), all ^1^H1–^1^H6 and the ^1^$H_{2}$O correlation signals were grouped together. Table 2 reveals that this net fractional intensity accounts for ≈42% of the entire 2D NMR signal and even more for the DFT (≈51%) and XRD (≈63%) structures. The remaining deviations also account for the significant variations in the $r_{\mathrm{eff}}^{\mathrm{NMR}}(H^{j}$–$H^{k})$ value for most $H^{j}$–$H^{k}$ pairs as evaluated against the respective XRD and DFT structures. The $H^{A}\cdots H^{B}$ distance is the overall shortest (as expected), followed by numerous distances involving water/aromatic and aromatic/aromatic proton pairs that range between 227 and 248 pm (Figure 5b). Here, the main improvements by DFT stem from an expansion of each O–H^A^/O–H^B^ bond length from 82/85 pm of the XRD structure to 99 pm for both bonds after DFT optimization, altogether expanding the ^1^$H^{A\cdots 1}H^{B}$ distance from 131 pm in the XRD structure to 157 pm in the DFT counterpart. These atom adjustments largely account for the markedly improved ^1^$H^{A}O^{1}H^{B}$ chemical-shift predictions by GIPAW/DFT (Table 1).

Likewise, slight adjustments of the CO$H^{+},$ N**H**, and C$H_{3}$ positions by the DFT calculations that accounted for the significantly improved $\delta_{H}$ values (Table 1) also underpin the better agreement of the effective CO$H^{+}\cdots$N**H** and N**H**⋯C$H_{3}$ distances to the NMR experiments. Here, the shortest CO$H^{+}\cdots$N**H** distance contracted from 344 pm in the XRD structure [40] to 329 pm upon DFT optimization (Figure 5b), while the N**H**⋯C$H_{3}$ distance contracted slightly by 3 pm. Nonetheless, although this minor structural alteration significantly improved the agreement with NMR, the slightly lower DFT-derived values of $f_{\mathrm{DFT}}$(N**H**⋯C$H_{3})=0.050$ than that of $f_{\mathrm{NMR}}$(N**H**⋯C$H_{3})=0.055$ (Table 2) imply that the “real” N**H**⋯C$H_{3}$ distance remains a few pm shorter than that of 217 pm in the DFT structure of Figure 5b and the corresponding $r_{\mathrm{eff}}^{\mathrm{DFT}}=216$ pm value shown in Table 2; see Section 2.5.2.

The ParaHCl structure is stabilized by H bonds involving paracetamol, water, and the chloride anion [40], encompassing the following bonds that appear in a sheet, shown in the structure fragment of Figure 5b: CO$H^{+}\cdots H_{2}O$ (144 pm); 1(O**H**)⋯${\mathrm{Cl}}^{-}$ (211 pm); N**H**⋯${\mathrm{Cl}}^{-}$ (225 pm). The ${\mathrm{Cl}}^{-}$ anions also form H bonds with each water proton, with the $H^{A}\cdots {\mathrm{Cl}}^{-}$ (211 pm) and $H^{B}\cdots {\mathrm{Cl}}^{-}$ (217 pm) bonds appearing within the sheet and interconnecting neighboring sheets, respectively. The DFT optimization relaxed the proton positions such that the H bond shortened and the proton became more centered between the two electronegative atoms. For example, the ≈15 pm O–H^A^/H^B^ bond’s lengthening (*vide supra*) was accompanied by a nearly equal*shortening* of the $H^{A}\cdots {\mathrm{Cl}}^{-}$ (228 pm) and $H^{B}\cdots {\mathrm{Cl}}^{-}$ (231 pm) distances encountered in the XRD structure [40].

#### 2.6.4. Results on ParaOA

Both XRD-derived ParaOA structures before and after DFT optimization overall agree well with the NMR experiments. Yet, as hinted from the slight *degradation* in the GIPAW/DFT-computed ^1^H chemical shifts from the DFT-optimized structure relative to its XRD counterpart (notwithstanding an *improved* match for the ^13^C shifts; Section 2.4), Table 2 reveals a slightly lower $R^{2}$(DFT)=0.96 correlation coefficient than that of $R^{2}$(XRD)=0.98 for the XRD structure. Hence, the ^1^H–^1^H distance analysis resolves the contradicting conclusions from the ^1^H and ^13^C chemical shift data, confirming an overall very similar quality of the DFT and XRD structures, yet with a slight preference of the latter. Although the rmsd of the effective-distance set associated with the DFT structure is slightly lower (16 pm) than that of the XRD structure (20 pm), it is evident from Table 2 and Figure 7 that this resulted from a significant improvement *only* for *two* long distances—C$H_{3}\cdots$**H**OA1, and the intramolecular **H**OA1⋯**H**OAN distance—both of which are markedly longer in the DFT-optimized structure—whereas *all* other ^1^H–^1^H distances reveal a nearly equal, or even worse, match with the NMR experiments as compared to the XRD structure, thereby accounting for the overall higher $R^{2}$ correlation coefficient for the XRD structure.

Note that the two intense C$H_{3}\cdots$H5/H6 and C$H_{3}\cdots$N**H** correlations partially overlap in the 2Q–1Q NMR spectrum of Figure 4c. They were grouped together and also omitted from the distance analysis because the observed NMR fractional intensity ($f_{\mathrm{NMR}}=0.39$) is substantially lower than those of both XRD/DFT structures (0.58/0.56). We have no convincing explanation for this observation. In order to avoid a large error propagation into all other distances, we proceeded by omitting that fractional intensity from normalization Equation (5). If included, a significant reduction is observed for both correlation coefficients, $R^{2}$(DFT)=0.87 and $R^{2}$(XRD)=0.90, but the conclusions remain, underscoring the robustness of the distance analysis for structural validations.

We now consider the paracetamol/OA intermolecular interactions evident in Figure 5c and confirmed by the 2Q–1Q NMR spectrum of Figure 4c. The relatively short 1(OH)⋯**H**OA1 effective distance stemming from the H-bonding of the 1(O**H**) and **H**OA1 moieties is overestimated and underestimated in the respective XRD and DFT structures to equal extents (8 pm). A close **H**2⋯**H**OA1 contact is also observed, which is underestimated slightly by both XRD and DFT structures relative to NMR, as is the effective C$H_{3}\cdots$**H**OAN distance, where Table 2 reveals that the DFT optimization produced a larger discrepancy (−24 pm) relative to the initial XRD structure (−17 pm).

## 3. Materials and Methods

### 3.1. Sample Preparation

Acetaminophen/paracetamol (Form I, monoclinic; 99% purity) and α-oxalic acid (98% purity) were purchased from Sigma-Aldrich (Stockholm, Sweden), along with concentrated hydrochloric acid (37%) and ethyl acetate (99.9%) obtained from Merck KGa (Darmstadt, Germany). Paracetamol hydrochloride monohydrate (ParaHCl) was prepared according to refs. [40,51]. Paracetamol (0.100 mol, 15.12 g) was immersed in 70 mL concentrated HCl and stirred at room temperature (RT) for 12 h. The resulting salt was isolated by vacuum filtration and dried in a desiccator. The paracetamol–oxalic-acid cocrystal (ParaOA) was obtained as in ref. [52] via slurry formation by adding 3.5 mL of ethyl acetate to stoichiometric amounts (50.0 mmol each) of paracetamol (7.56 g) and α-OA (4.50 g) at RT under continuous stirring for 48 h. The resulting cocrystal was isolated by vacuum filtration and dried in a desiccator.

### 3.2. X-Ray Powder Diffraction

Powder XRD (PXRD) patterns were collected from the Para, ParaHCl, and ParaOA specimens using a Bruker D8 Discover diffractometer (Bruker, Bremen, Germany) equipped with a LYNXEYE position-sensitive detector, using Cu $K\alpha_{1,2}$ radiation ($\lambda_{1}$ = 154.06 pm; $\lambda_{2}$ = 154.44 pm) and Bragg–Brentano geometry with sample spinning. Each powder was loaded on zero-background Si plates, and all diffractograms were collected at RT over a 2θ range of 5°–50°, employing a step size of 0.018° with 1.00 s per step, giving a total measurement time of ≈43 min per sample, respectively. Rietveld refinements were performed with the TOPAS software [53]. Figure S1 shows the diffractograms along with the Rietveld refinements.

No impurities were detected in the commercial Para specimen, whereas the ParaOA co-crystal was found to comprise 3.4 wt% of α-oxalic acid dihydrate (α-OA$\cdot 2H_{2}$O), which readily forms from the hygroscopic α-OA precursor; also see ref. [43]. As discussed in Section 2.1, however, essentially all of the α-OA$\cdot 2H_{2}$O appeared to have transformed into the metastable β-OA phase during fast-MAS NMR experimentation. The as-prepared ParaHCl salt was also phase-pure but is unstable under ambient conditions and readily transforms into bis(acetaminophen) hydrochloride, abbreviated here as bis(Para)HCl and also referred to as acetaminophen hemihydrochloride [40,54]. Although a shorter PXRD experiment (8 min) over the same 2θ range but a 0.02° step size and 0.2 s per step indeed evidenced a phase-pure ParaHCl sample, the ParaHCl→bis(Para)HCl transformation was detected (3.6 wt%) from the longer PXRD experiment described above, as well as during MAS NMR experimentation.

### 3.3. Solid State NMR

All solid-state NMR experiments were performed at ambient temperature using a Bruker Avance-III spectrometer (Bruker, Rheinstetten, Germany) at a magnetic field of 14.1 T that produces Larmor frequencies of −600.12 MHz for ^1^H and −150.91 MHz for ^13^C. 1.3 and 4.0 mm zirconia rotors were filled with powders of the Para, ParaHCl, and ParaOA specimens. Except for the ^1^H$\to^{13}$C CPMAS NMR experiments that were performed with a 4 mm MAS probehead, all experiments utilized a 1.3 mm probehead and rotors undergoing MAS at the rate $\nu_{r}=60.00$ kHz. Resonance offsets were minimized by positioning each radio-frequency (rf) carrier ^1^H/^13^C frequency at the center of the resonance region throughout. ^1^H and ^13^C chemical shifts are quoted relative to neat tetramethylsilane (TMS).

Quantitative Bloch-decay ^1^H NMR spectra were recorded with 90° radio-frequency (rf) pulses at the nutation frequency of $\nu_{H}=104$ kHz. Relaxation delays ($\tau_{\mathrm{relax}}$) of {160, 15, 200} s and {16, 8, 4} accumulated NMR-signal transients were acquired for {Para, ParaHCl, ParaOA}, respectively. Background signals from the NMR probehead/rotor were eliminated by subtracting the result from a separately acquired NMR spectrum from the empty rotor but with otherwise identical experimental parameters.

^13^C NMR spectra were recorded at $\nu_{r}=12.00$ kHz by ^1^H$\to^{13}$C CPMAS under the modified Hartmann–Hahn condition $\nu_{C}-\nu_{H}=\nu_{r}$, with a contact period of $\tau_{\mathrm{CP}}=1.25$ ms, $\nu_{H}=35$ kHz, and a linearly ramped [55] rf amplitude for ^13^C, i.e., $\nu_{C}=46\pm 3\;$kHz. Throughout the experiment, the 90° ^1^H pulse prior to CP operated at $\nu_{H}\approx 100$ kHz and SPINAL-64 [56] proton decoupling at $\nu_{H}\approx$ 78 kHz was applied during ^13^C NMR-signal detection. The {512, 512, 1024} signal transients were acquired for the respective {Para, ParaHCl, ParaOA} specimens, with 15 s relaxation delays for Para and ParaOA and 30 s for ParaHCl.

### 3.4. 2D NMR Experiments

2Q–1Q ^1^H NMR correlation spectra were recorded with the 2D NMR protocol shown in Figure 1a of ref. [45]. The 2Q coherence (2QC) excitation/reconversion was accomplished by the shortest possible BaBa dipolar recoupling rf-pulse scheme, which extends over one sole rotor period [45,50], thereby giving 2Q excitation ($\tau_{\mathrm{exc}}$) and reconversion ($\tau_{\mathrm{rec}}$) intervals of $\tau_{\mathrm{exc}}=$ $\tau_{\mathrm{exc}}=\tau_{r}=16.67$ µs, where $\tau_{r}=$$\nu_{r}^{-1}$ is the rotor period. The ^1^H nutation frequency was $\nu_{H}=156$ kHz for the 90° dipolar recoupling pulses of duration 1.60 µs. All 2D NMR acquisitions employed a spectral window of 32 kHz in the direct spectral dimension and rotor-synchronized 2QC evolution with $\Delta t_{1}=$ {$3\tau_{r},$ $4\tau_{r},2\tau_{r}$} for {Para, ParaHCl, ParaOA}, respectively, along with {40, 66, 40} $t_{1}$ data points recorded and relaxation delays of {75, 15, 75} s. Note that these much shorter $\tau_{\mathrm{relax}}$ values used for the 2Q–1Q ^1^H NMR experiments relative to the Bloch-decay counterparts were required for practical reasons; we verified the absence of any significant differential relaxation among the various proton sites. The 2D datasets were zero-filled to 128 × 4096 ($t_{1},$ $t_{2}$) points for the experiments on Para and ParaHCl, and they were zero-filled to 256 × 4096 for ParaOA and apodized with 100 Hz Gaussian and 50 Hz Lorentzian broadening along the indirect and direct dimensions, respectively. For achieving absorptive 2D NMR peaks with frequency-sign discrimination along the indirect dimension, all 2D NMR acquisitions implemented the States procedure [57]. The 2D NMR spectra are shown with a lowest contour level of ≈2%.

A ^13^${\mathrm{C\{}}^{1}$H} 2D HETCOR NMR experiment was performed on the ParaHCl sample, employing ^1^H$\to^{13}$C CP for $\tau_{\mathrm{CP}}=250$ µs established at the Hartmann–Hahn condition [58], $\nu_{C}-\nu_{H}=\nu_{r}$, using $\nu_{H}=20$ kHz and a ramped CP around $\nu_{C}=80\pm 13\;$kHz and a 2.4 µs 90° ^1^H pulse. The HETCOR NMR spectrum was recorded with $\tau_{\mathrm{relax}}=16\;$s, and spectral windows of 65 kHz and 25 kHz were used in the ^13^C ($t_{2}$) and ^1^H ($t_{1}$) dimensions. The $50t_{1}$ values were collected with 96 accumulated transients per $t_{1}$ value and SPINAL-64 proton decoupling at $\nu_{H}=$145 kHz during $t_{2}$. The 2D NMR datasets were zero-filled to 1024 × 4096 ($t_{1},$ $t_{2}$) points and apodized by 200 and 50 Hz Lorentzian broadening along the $t_{1}$ and $t_{2}$ dimensions, respectively.

### 3.5. GIPAW/DFT Calculations

Geometry optimizations by first-principles DFT calculations were carried out with the CASTEP software (version 22.11) [59] using the XRD-derived unit-cell parameters reported in the literature [39,40,41]. The Perdew–Burke–Ernzerhof (PBE) functional [60] was employed with on-the-fly generated ultrasoft pseudopotentials [61] and a plane-wave basis set [62]. The Grimme D3 scheme with Becke-Johnson damping [63] was used for the dispersion correction. The XRD-derived Para (Form I, monoclinic; CCDC 754966) [41] and ParaHCl (monoclinic, CCDC 835705) [40] structures were refined by only adjusting the H atom positions and using fixed coordinates of all heavier atoms. Whereas no improvements resulted by also adjusting the coordinates of the latter atoms of those structures, that of ParaOA (monoclinic; CCDC 720368) [39] improved due to the all-atom refinements. Indeed, as opposed to the Para and ParaHCl structures, the (only available) reported ParaOA structure was derived from PXRD data, whose lower accuracy than single-crystal XRD results might involve less accurate cell parameters.

The chemical shielding values for ^1^H and ^13^C were calculated for all XRD structures before and after optimization using the GIPAW method [17,18,19,20,21,22]. For both DFT geometry optimizations and the GIPAW magnetic shielding parameter calculations, a plane-wave energy cut-off of 1000 eV was used along with a Monkhorst–Pack *k*-point grid and a maximum spacing of 0.05 ${Å}^{-1}$ in the reciprocal space.

We utilize the chemical shift scale throughout, where low (high) chemical shifts correspond to shielded (deshielded) nuclei, and the *isotropic chemical shift* is given by [64,65,66]

(12) $$\delta_{S}^{j}\equiv \delta_{S}^{\mathrm{iso},j}=\frac{1}{3}\left(\delta_{xx}^{Sj}+\delta_{yy}^{Sj}+\delta_{zz}^{Sj}\right)\;\mathrm{for}\;S={\{}^{1}H{,}^{13}C\}.$$

The GIPAW/DFT-derived principal values of the chemical shift tensor of spin-species S, {$\delta_{xx}^{Sj}$, $\delta_{yy}^{Sj}$, $\delta_{zz}^{Sj}$}, were calculated from the respective magnetic shielding values, {$\sigma_{xx}^{Sj}$, $\sigma_{yy}^{Sj}$, $\sigma_{zz}^{Sj}$}, according to [20,21,22]

(13) $$\delta_{\alpha \alpha }^{Sj}=\sigma_{\mathrm{ref}}^{S}-\sigma_{\alpha \alpha }^{Sj}\;,\;\mathrm{with}\;\alpha \alpha =\left\{xx,\;yy,\;zz\right\}\;\mathrm{and}\;S={\{}^{1}H{,}^{13}C\}.$$

All three structures employed the shielding-to-shift conversion terms $\sigma_{\mathrm{ref}}^{H}=30.44$ ppm and $\sigma_{\mathrm{ref}}^{C}=171.70$ ppm. Both were obtained from the intercept that minimized the difference between the experimental and calculated {$\delta_{H}^{j}$} or {$\delta_{C}^{j}$} values for the Para structure, yielding correlation coefficients of $R^{2}=0.983$ and $R^{2}=0.998$ for ^1^H and ^13^C, respectively.

## 4. Conclusions

By coupling DFT calculations with 1D and 2D MAS NMR experiments, the ^1^H and ^13^C chemical shifts and NMR-peak assignments were extracted from monoclinic paracetamol (Para), along with its HCl monohydrate salt (ParaHCl) and cocrystal with oxalic acid (ParaOA). The chemical shifts and their site assignments are, to our knowledge, reported for the first time, while those from Para were in excellent agreement with an earlier study [46]. Each original XRD-derived Para [41], ParaHCl [40], and ParaOA [39] crystal structure was refined by plane-wave DFT calculations. The GIPAW/DFT ^1^H and ^13^C chemical-shift predictions before and after geometry optimization were contrasted with the experimental shifts from MAS NMR. These evaluations revealed excellent agreement for the original Para structure, with only very minor improvements after geometry optimization by DFT, while the chemical shifts predicted from the XRD-derived ParaHCl structure exhibited poor agreement with the NMR shifts, which improved substantially upon DFT optimization. For ParaOA, on the other hand, the chemical-shift evaluations were inconclusive for the DFT and XRD structures, with a slightly worse (better) agreement for the $\delta_{H}$ ($\delta_{C}$) values of the DFT-optimized structure with experiments.

The chemical-shift structural assessment protocol utilized herein has been well established for more than two decades [19,20,21,22,23]. While being straightforward, it does not involve direct distance constraints in the evaluation compared to that of the analysis of the fractional integrated 2D NMR spectral intensities of the 2Q–1Q correlation spectrum [Equation (4)]. The latter may be directly evaluated against the respective fractional effective dipolar coupling constants calculated from the structure model to be evaluated via Equations (3) and (6), thereby yielding a single figure of merit (i.e., $R^{2}$) on the quality of agreement. This analysis procedure is markedly less effort-intensive than earlier options for analyzing dipolar interactions by 2D correlation NMR, which required recording several 2D NMR spectra with increasing dipolar recoupling intervals, integrating all peak intensities and then contrasting the 2D NMR-signal buildup with results from either computationally demanding multi-spin simulations [30,31,32,33] or phenomenological, and thereby approximate, expressions [27,28,29]. We note that although not utilized herein, the procedure of Section 2.5 is also readily applied to heteronuclear 2D correlation NMR spectra [37,38], such as for assessing ^1^H–^13^C distances.

Although the direct comparison of fractional intensities/effective dipolar coupling constants is sufficient for gauging the quality of the evaluated structure model, the effective dipolar interaction may also be converted into an ^1^$H^{j}$–^1^H^*k*^ “effective distance” [Equation (8)], which is an aggregate property with a value close to the shortest ^1^H^*j*^–^1^H^*k*^ distance in the structure. Here, the deviation between the effective distance calculated from the reference structure and its NMR-derived counterpart conveys the extent of the over/under-estimation of the ^1^H^*j*^–^1^H^*k*^ distance in the reference structure. The main analysis obstacle is insufficient 2D NMR spectral resolution, which can nevertheless be mitigated by grouping H sites together in the structural analysis, thereby still enabling analysis but at the cost of reduced statistics underlying the $R^{2}$ correlation coefficient.

For our present structural assessments of the three paracetamol-derived structures, the distance-based analysis gave essentially identical conclusions as the validation based on chemical shifts, except for the ParaOA structure, where the distance analysis confirmed very similar qualities of the DFT and XRD structures yet with a slight preference of the latter structure. We attribute this unexpected result to the structural derivation from powder XRD data, which, despite an all-atom refinement by DFT, did not improve results, likely owing to inaccurate cell parameters that were not optimized by DFT. Moreover, despite significant improvement by the DFT optimization of the XRD-derived ParaHCl structure and an excellent DFT/NMR agreement in the ^1^H chemical shifts (rmsd = 0.18 ppm), the rather low correlation coefficient ($R^{2}=0.90$) of the distance analysis suggests remaining discrepancies with respect to the 2D NMR distance constraints, of which we have no explanation. Besides overall supporting the validity of contrasting calculated and experimental chemical shifts, the 2Q–1Q ^1^H NMR analysis provides more direct insight into the differences in ^1^H–^1^H distances between the model structure (here, from XRD) and the NMR constraints.

## Figures and Tables

**Table 1:** ^1^H and and DFT calculations ^**a**^.

	^**1**^H Chemical Shift (ppm)			^**13**^C Chemical Shift (ppm)		
**Site**	**NMR**	**DFT($\Delta_{H}^{j}$)**	**XRD($\Delta_{H}^{j}$)**	**NMR**	**DFT($\Delta_{C}^{j}$)**	**XRD($\Delta_{C}^{j}$)**
**Para**						
1 (OH)	9.2	9.79 (0.59)	9.32 (0.12)	152.18	154.21 (2.03)	154.06 (1.88)
2	5.75	5.42 (−0.33)	5.31 (−0.44)	116.19	116.56 (0.37)	115.82 (−0.37)
3	* 6.6	6.76 (0.16)	6.67 (0.07)	120.48	121.49 (1.01)	121.66 (1.18)
4				132.91	133.33 (0.42)	134.26 (1.36)
5	7.86	7.95 (0.09)	7.90 (0.04)	123.24	124.62 (1.38)	124.36 (1.12)
6	* 6.6	6.58 (−0.02)	6.48 (−0.12)	115.64	115.82 (0.18)	115.78 (0.14)
CO				169.69	166.50 (−3.19)	165.97 (−3.72)
NH	9.03	9.47 (0.44)	9.06 (0.04)			
${\mathrm{CH}}_{3}$	1.07	0.68 (−0.40)	0.20 (−0.88)	23.64	21.43 (−2.21)	18.07 (−5.57)
**rmsd (ppm)**		**0.35**	**0.38**		**1.67**	**2.57**
**ParaHCl**						
1 (OH)	8.2	8.33 (0.13)	−0.99 (−9.19)	155.79	157.66 (1.87)	157.55 (1.75)
2	* 6.5	6.58 (0.08)	4.24 (−2.26)	114.66	113.92 (−0.74)	107.11 (−7.54)
3	6.7	6.62 (−0.08)	5.00 (−1.70)	* 122.23	123.12 (0.89)	117.79 (−4.44)
4				128.22	128.85 (0.63)	128.03 (−0.19)
5	* 6.5	6.46 (−0.04)	4.01 (−2.49)	* 122.23	122.17 (−0.06)	116.03 (−6.20)
6	6.2	6.17 (−0.03)	3.36 (−2.84)	117.54	118.07 (0.53)	113.47 (−4.07)
${\mathrm{COH}}^{+}$	16.91	17.22 (0.31)	10.86 (−6.04)	172.43	168.37 (−4.07)	169.89 (−2.55)
NH	10.76	10.39 (−0.37)	4.24 (−6.52)			
${\mathrm{CH}}_{3}$	2.82	2.56 (−0.26)	−1.84 (−4.67)	22.63	19.62 (−3.01)	−2.00 (−24.62)
$H_{2}^{A}$O	7.2	7.21 (0.01)	−2.23 (−9.43)			
$H_{2}^{B}$O	6.6	6.58 (−0.02)	−0.97 (−7.57)			
**rmsd (ppm)**		**0.18**	**5.95**		**1.97**	**9.67**
**ParaOA**						
1 (OH)	8.3	9.23 (0.93)	8.92 (0.62)	150.78	154.60 (3.82)	152.61 (1.83)
2	5.5	5.54 (0.04)	5.31 (−0.19)	* 115.05	115.41 (0.35)	110.81 (−4.25)
3	6.5	6.66 (0.16)	6.42 (−0.08)	* 120.86	122.50 (1.65)	121.49 (0.64)
4				132.77	133.74 (0.97)	134.02 (1.24)
5	* 7.2	7.39 (0.19)	7.37 (0.17)	* 120.86	123.26 (2.41)	123.06 (2.21)
6	* 7.2	7.18 (−0.02)	6.85 (−0.35)	* 115.05	115.03 (−0.02)	117.34 (2.29)
NH	9.3	10.17 (0.87)	10.00 (0.70)			
CO				173.84	173.32 (−0.52)	171.12 (−2.72)
${\mathrm{CH}}_{3}$	1.99	1.78 (−0.21)	1.46 (−0.53)	24.27	21.80 (−2.47)	19.92 (−4.35)
HOA1	11.68	12.57 (0.90)	11.06 (−0.62)	160.38	162.79 (2.40)	162.01 (1.62)
HOAN	14.75	16.39 (1.64)	13.62 (−1.13)	159.43	161.47 (2.04)	158.85 (−0.58)
**rmsd (ppm)**		**0.76**	**0.58**		**2.01**	**2.50**

^**a**^ Chemical shifts obtained from NMR or computed by GIPAW/DFT on XRD-derived structures before (“XRD”) and after (“DFT”) geometry optimization by DFT (Section 2.4). The values within parentheses represent the deviation between the GIPAW/DFT predictions and the experimental chemical shift [Equation (1)]. The root mean square deviation (rmsd) between calculated and experimental chemical-shift sets are given for each structure (bold-face numbers). Values marked by asterisks involve sites with completely overlapping MAS NMR peaks, for which the chemical shift is taken as the peak maximum.

**Table 2:** Effective ^1^H–^1^H distances ^**a**^.

Sites		$f_{\mathrm{NMR}}$	$f_{\mathrm{DFT}}$	$f_{\mathrm{XRD}}$	$r_{\mathrm{eff}}^{\mathrm{DFT}}\left(r_{\mathrm{eff}}^{\mathrm{NMR}}\right)$	$\Delta r_{\mathrm{eff}}^{\mathrm{DFT}}$	$r_{\mathrm{eff}}^{\mathrm{XRD}}\left(r_{\mathrm{eff}}^{\mathrm{NMR}}\right)$	$\Delta r_{\mathrm{eff}}^{\mathrm{XRD}}$
					**(pm)**	**(pm)**	**(pm)**	**(pm)**
**Para**								
1 (O**H**),N**H**								
	H2	0.029	0.024	0.021	229 (220)	9	230 (218)	12
	H3,H6	0.071	0.061	0.057	235 (227)	8	235 (226)	9
	C$H_{3}$	0.074	0.067	0.060	193 (188)	5	194 (187)	7
H2								
	H3,H6	0.034	0.019	0.018	237 (214)	23	236 (213)	23
	C$H_{3}$	0.056	0.051	0.043	202 (197)	5	205 (196)	9
H3,H6								
	H5	0.022	0.022	0.020	232 (230)	2	233 (229)	4
	C$H_{3}$	0.065	0.039	0.035	211 (192)	19	211 (191)	20
H5								
	H5	0.026	0.030	0.026	221 (224)	−3	222 (222)	0
	C$H_{3}$	0.012	0.012	0.011	290 (287)	3	290 (285)	5
C$H_{3}$								
	CH_3_^**b**^	0.611	0.675	0.709	177 (178)	−1	173 (177)	−4
***R*** ^**2**^ **/rmsd (pm)**			**0.980**	**0.976**		**10.5**		**11.5**
**ParaHCl**								
1–6,$H_{2}$O								
	1–6,$H_{2}$O	0.424	0.506	0.632	164 (169)	−5	144 (154)	−10
	CO$H^{+}$	0.085	0.141	0.078	203 (221)	−18	205 (202)	3
	N**H**	0.113	0.054	0.033	213 (188)	25	211 (172)	39
	C$H_{3}$	0.122	0.098	0.055	260 (251)	9	260 (228)	32
CO$H^{+}$								
	N**H**	0.010	0.005	0.003	318 (301)	17	315 (273)	42
	C$H_{3}$	0.032	0.039	0.026	225 (236)	−11	219 (214)	5
N**H**								
	C$H_{3}$	0.055	0.050	0.030	216 (212)	4	213 (193)	20
C$H_{3}$								
	CH_3_^**c**^	0.159	0.107	0.143	181 (170)	11	157 (154)	3
***R*** ^**2**^ **/rmsd (pm)**			**0.900**	**0.823**		**14.1**		**24.7**
**ParaOA**								
1–6,N**H**								
	1–6,N**H**	0.458	0.384	0.404	230 (223)	7	222 (218)	4
H5,H6,N**H**								
	CH_3_^**d**^	0.386	0.578	0.564				
H5,H6								
	**H**OAN	0.035	0.047	0.044	314 (330)	−16	305 (317)	−12
C$H_{3}$								
	CH_3_^**c**^	0.295	0.274	0.294	177 (175)	2	168 (168)	0
	**H**OA1	0.010	0.017	0.020	332 (364)	−32	314 (354)	−40
	**H**OAN	0.063	0.112	0.094	242 (266)	−24	242 (259)	−17
**H**OA1								
	1 (O**H**)	0.080	0.099	0.065	220 (228)	−8	227 (219)	8
	H2	0.049	0.054	0.057	243 (247)	−4	232 (238)	−6
	**H**OAN	0.010	0.013	0.022	309 (321)	−12	276 (312)	−36
***R*** ^**2**^ **/rmsd (pm)**			**0.966**	**0.983**		**16.5**		**20.9**

^**a**^ Fractional 2Q–1Q NMR intensities [$f_{\mathrm{NMR}}$; Equation (4)] and the counterparts $f_{\mathrm{XRD}}$ and $f_{\mathrm{DFT}}$ calculated from Equation (6) and the atom coordinates in the XRD structure before and after DFT optimization, along with the effective ^1^H–^1^H distances extracted from Equation (8), $r_{\mathrm{eff}}^{\alpha }$ with α={NMR, XRD, DFT}. $\Delta r_{\mathrm{eff}}^{\mathrm{XRD}}$($H^{j}$–$H^{k}$) and $\Delta r_{\mathrm{eff}}^{\mathrm{DFT}}$($H^{j}$–$H^{k}$) were calculated from Equation (11), whereas the correlation coefficient ($R^{2}$) of the agreement between the set {$f_{\mathrm{NMR}}$} and the XRD/DFT counterparts was evaluated from Equation (10), as well as the rmsd (in pm) between each {$r_{\mathrm{eff}}^{\mathrm{XRD}}$} and {$r_{\mathrm{eff}}^{\mathrm{DFT}}$} set to {$r_{\mathrm{eff}}^{\mathrm{NMR}}$}. ^**b**^ Assumed absence of rotation around the $C_{3}$ axis. ^**c**^ Assumed rapid rotation around the $C_{3}$ axis; see Section 2.6.2. ^**d**^ These values were excluded from the distance analysis; see Section 2.6.4.

## Data Availability

Raw data are accessible from the following link: https://su.drive.sunet.se/s/DLgf9DKknAYtTZi (accessed on 28 April 2026).

## Supplementary Materials

The following supporting information can be downloaded at https://www.mdpi.com/article/10.3390/molecules31101584/s1. Figure S1: Powder X-ray diffractograms of cocrystals and results from Rietveld refinements. Figure S2: ${\{}^{1}{\mathrm{H\}}}^{13}$C HETCOR spectrum of ParaHCl. Table S1: Unit-cell parameters from Rietveld refinements.
