# Supplementary material for: 1H–1H Interatomic Distances in Paracetamol-Based Structures Unveiled by Double-Quantum NMR and DFT Calculations
Source: Molecules. 2026 May 9;31(10):1584. doi: 10.3390/molecules31101584 (PMC13210120; doi:10.3390/molecules31101584)
Supplement: Supplementary file 1 [file molecules-31-01584-s001.zip › molecules-4296051-supplementary.pdf]

# Supporting Information for
# $^1$H–$^1$H Interatomic Distances in Paracetamol-Based Structures Unveiled by Double-Quantum NMR and DFT Calculations

Martins Balodis†, Baltzar Stevensson, Debashis Majhi ‡, Tra Mi Nguyen, Chaithanya Hareendran, and Mattias Edén*

Department of Chemistry, Stockholm University, SE-106 91 Stockholm, Sweden; martins.balodis@osi.lv (M.B.);

baltzar.stevensson@su.se (B.S.); debashis@nitt.edu (D.M.); trantrami.nguyen@su.se (T.M.N.);

chaithanya.hareendran@su.se (C.H.)

* Correspondence: mattias.eden@su.se

† Current address: Laboratory of Physical Chemistry, Latvian Institute of Organic Synthesis, 1006 Riga, Latvia.

‡ Current address: Department of Chemistry, National Institute of Technology Tiruchirappalli, Tiruchirappalli 620015, India.

## Contents

1. **Figure S1.** Powder X-Ray Diffractograms of Cocrystals and Results from Rietveld Refinements.
2. **Figure S2.** $\{^1H\}^{13}C$ HETCOR Spectrum of ParaHCl.
3. **Table S1.** Unit-Cell Parameters from Rietveld Refinements.
4. **References.**

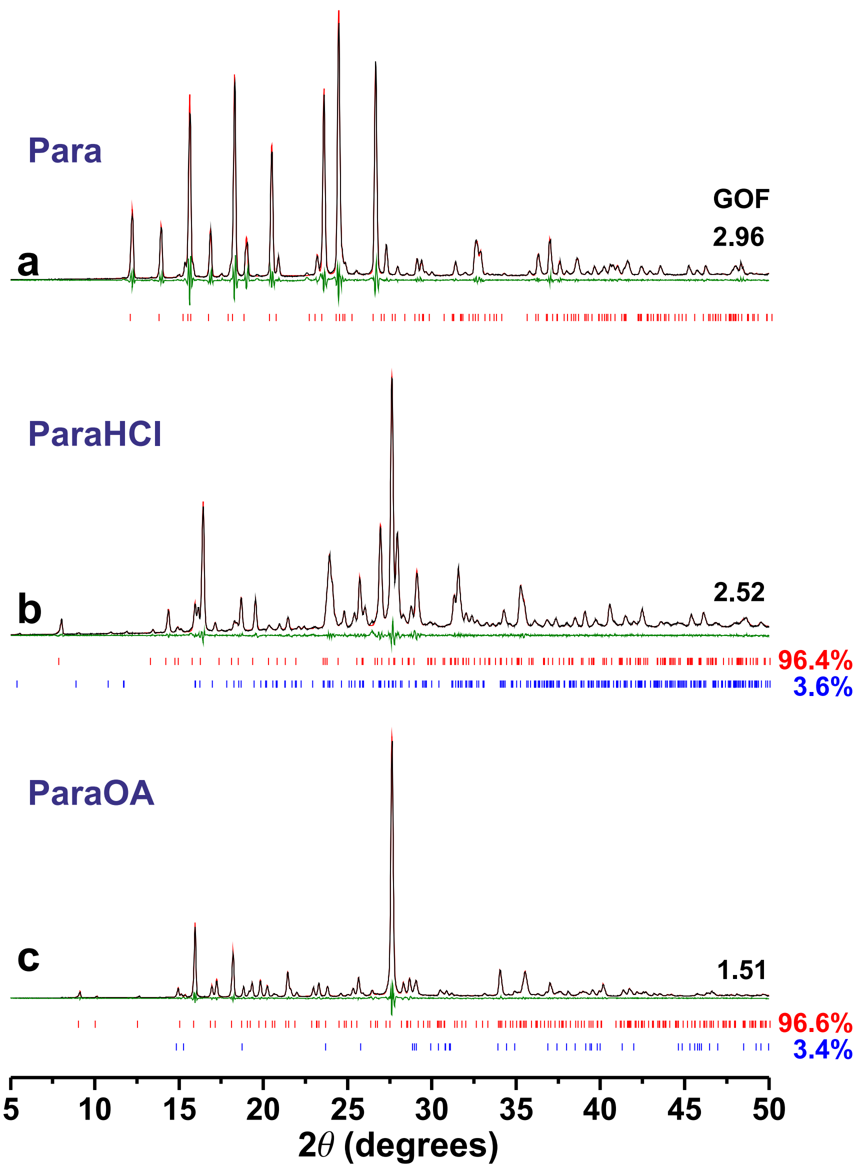


**Figure S1.** Experimental powder X-ray diffractograms (black traces) and calculated best-fit profiles (red traces) obtained by the Rietveld method for the (a) Para, (b) ParaHCl, and (c) ParaOA specimens. Each number shown on the right portion of the diffractogram is the "goodness-of-fit"(GOF),[S1] while the green curve beneath each PXRD pattern represents the difference between the experimental data and the best fit. The ParaHCl and ParaOA powders comprised minor amounts of bis(acetaminophen) hydrochloride [bis(Para)HCl] and $\alpha$-oxalic acid dihydrate ($\alpha$-OA·2$H_2$O), respectively. The relative phase compositions (in %) are given to the right of the respective Bragg-peak markers, which represent the following: (a) Para (red, monoclinic, space group $P2_1/n$; CCDC 754966),[S2] (b) ParaHCl (red, monoclinic, space group $P2_1/c$; CCDC 835705)[S3] and bis(Para)HCl (blue, monoclinic, space group $P2_1/c$; CCDC 835707);[S4] (c) ParaOA (red, monoclinic, space group $P2_1/c$; CCDC 720368)[S5] and $\alpha$-OA·2$H_2$O (blue, monoclinic, space group $P2_1/n$; CCDC 703783).[S6] The cell parameters of each structure before and after Rietveld refinement are presented in Table **S1**.

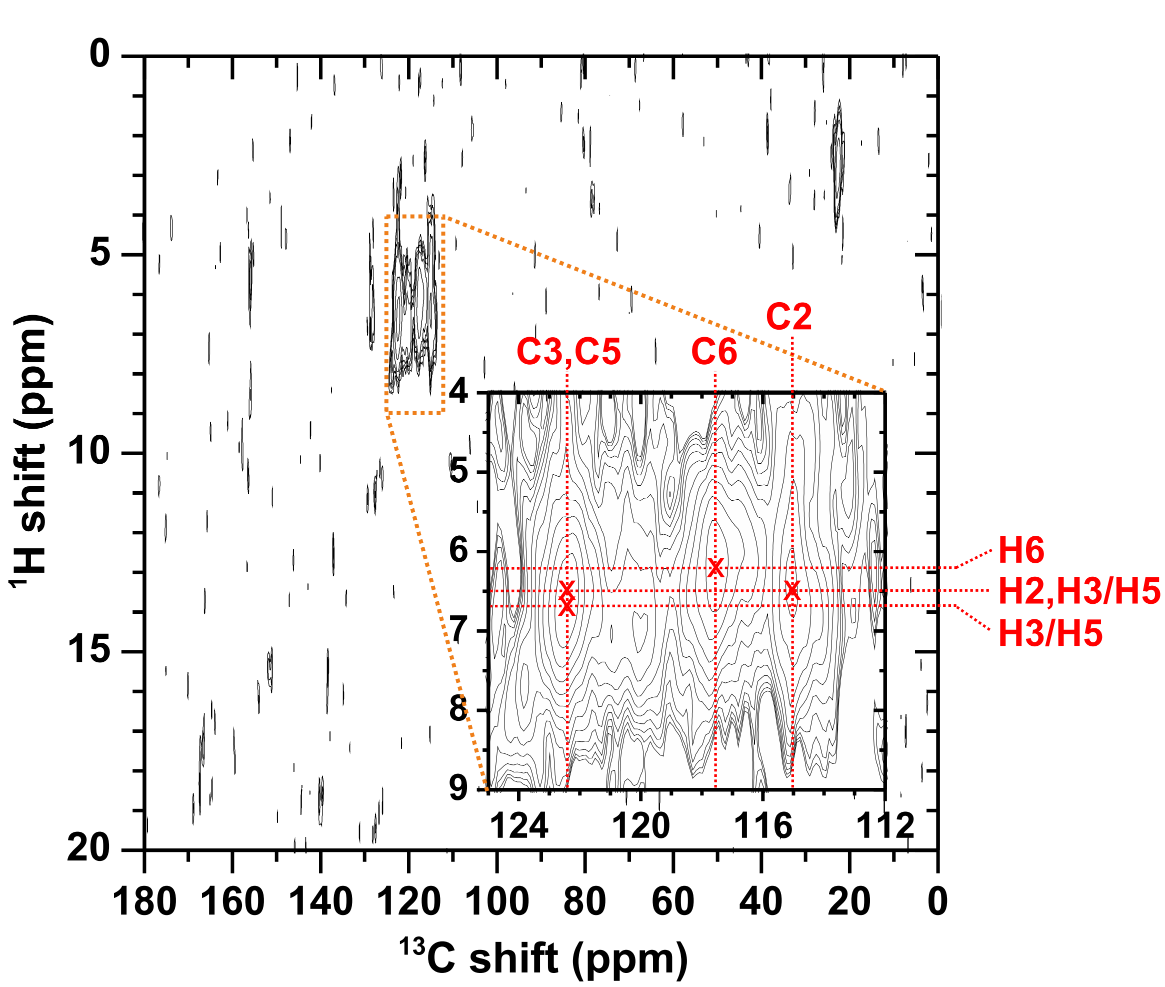


**Figure S2.** $\{^1H\}^{13}C$ HETCOR NMR spectrum recorded from ParaHCl at $B_0 = 14.1$ T and $\nu_r = 60.00$ kHz MAS, and obtained with a $^1H \rightarrow ^{13}C$ CP contact time period of 250 $\mu$s. The inset spectral portion is a zoom of the aromatic region, which assisted the $^1H$ NMR peak assignment of the H2, H3, H5 and H6 sites.

**Table S1. Unit-Cell Parameters by Rietveld Fitting of XRD Data[a]**

| | Cell lengths (pm) | | | Cell angles (degree) | | | | | |
|---|---|---|---|---|---|---|---|---|---|
| Phase | $a$ | $b$ | $c$ | $\alpha$ | $\beta$ | $\gamma$ | Space group | CCDC[b] | Ref. |
| Para | 710.81 | 939.81 | 1172.40 | 90.0000 | 97.4317 | 90.0000 | $P2_1/n$ | | this work |
| | 709.07 | 921.14 | 1159.72 | 90.0000 | 97.8440 | 90.0000 | $P2_1/n$ | 754966 | S2 |
| ParaHCl | 629.14 | 2243.77 | 703.80 | 90.0000 | 98.2876 | 90.0000 | $P2_1/c$ | | this work |
| | 627.61 | 2234.50 | 688.33 | 90.0000 | 97.2570 | 90.0000 | $P2_1/c$ | 835705 | S3 |
| bis(Para)HCl | 1717.15 | 500.29 | 2086.71 | 90.0000 | 107.5498 | 90.0000 | $P2_1/c$ | | this work |
| | 1713.98 | 484.96 | 2099.12 | 90.0000 | 108.3060 | 90.0000 | $P2_1/c$ | 835707 | S4 |
| ParaOA | 518.50 | 1177.85 | 1767.40 | 90.0000 | 94.2342 | 90.0000 | $P2_1/c$ | | this work |
| | 516.38 | 1173.23 | 1759.34 | 90.0000 | 94.3285 | 90.0000 | $P2_1/c$ | 720368 | S5 |
| $\alpha$-OA·2$H_2O$ | 612.58 | 361.54 | 1208.46 | 90.0000 | 106.3321 | 90.0000 | $P2_1/n$ | | this work |
| | 611.69 | 360.53 | 1204.90 | 90.0000 | 106.3030 | 90.0000 | $P2_1/n$ | 703783 | S6 |

[a] Unit-cell parameters obtained from Rietveld refinement of the PXRD data shown in Fig. **S1** and using the TOPAS software[S1] (first line of each entry), along with the previously reported input parameters (second line) from the as-stated source (rightmost column).
[b] Deposition number in the Cambridge crystallographic data center.

# References


S1 A. Coelho, TOPAS-academic v6, *Coelho Software*, 2016.

S2 Y. V. Nelyubina, I. V. Glukhov, M. Y. Antipin, M. Yu., and K. A. Lyssenko, "Higher density does not mean higher stability" mystery of paracetamol finally unraveled, *Chem. Commun.*, 2010, **46**, 3469–3471.

S3 S. R. Perumalla, L. Shi, and C. C. Sun, Ionized form of acetaminophen with improved compaction properties, *CrystEngComm*, 2012, **14**, 2389–2390.

S4 S. R. Perumalla and C. C. Sun, Confused hcl: Hydrogen chloride or hydrochloric acid?, *Chem. Eur. J.*, 2012, **18**, 6462–6464.

S5 S. Karki, T. Friščić, L. Fábián, P. R. Laity, G. M. Day, and W. Jones, Improving mechanical properties of crystalline solids by cocrystal formation: New compressible forms of paracetamol, *Adv. Mater.*, 2009, **21**, 3905–3909.

S6 N. Casati, P. Macchi, and A. Sironi, Hydrogen migration in oxalic acid di-hydrate at high pressure?, *Chem. Commun.*, 2009, 2679–2681.
